# The Biological and Anthropogenic Soundscape of an Urbanized Port – the Charleston Harbor Estuary, South Carolina, USA

**DOI:** 10.1371/journal.pone.0283848

**Published:** 2023-04-19

**Authors:** Lindsey Transue, Agnieszka Monczak, Caroline Tribble, Alyssa Marian, Patricia Fair, Joseph Ballenger, Brian Balmer, Eric W. Montie

**Affiliations:** 1 Department of Natural Sciences, University of South Carolina Beaufort, Bluffton, SC, United States of America; 2 Graduate Program in Marine Biology, College of Charleston, Charleston, SC, United States of America; 3 Institute of Biological and Environmental Sciences, University of Aberdeen, Aberdeen, United Kingdom; 4 Depart. of Public Health Science, Medical University of South Carolina, Charleston, SC, United States of America; 5 South Carolina Aquarium, Charleston, SC, United States of America; 6 South Carolina Department of Natural Resources, Charleston, SC, United States of America; 7 Dolphin Relief and Research, Clancy, MT, United States of America; DePaul University, UNITED STATES

## Abstract

Soundscape ecology provides a long-term, noninvasive approach to track animal behavior, habitat quality, and community structure over temporal and spatial scales. Using soniferous species as an indicator, biological soundscapes provide information about species and ecosystem health as well as their response and resiliency to potential stressors such as noise pollution. Charleston Harbor, South Carolina, USA provides important estuarine habitat for an abundance of marine life and is one of the busiest and fastest growing container ports in the southeast USA. Six passive acoustic recorders were deployed in the Charleston Harbor from December 2017 to June 2019 to determine biological patterns and human-associated influences on the soundscape. Anthropogenic noise was detected frequently across the estuary, especially along the shipping channel. Despite this anthropogenic noise, biological sound patterns were identified including snapping shrimp snaps (*Alpheus spp*. and *Synalpheus spp*.), fish calling and chorusing (Sciaenidae and Batrachoididae families), and bottlenose dolphin vocalizations. Biological response to anthropogenic activity varied among trophic levels, with decreased detection of fish calling when anthropogenic noise occurred and increased dolphin vocalizations in the presence of anthropogenic noise. Statistically, fine-scale, temporal patterns in biological sound were not clearly identified by sound pressure levels (SPLs), until files with anthropogenic noise presence were removed. These findings indicate that SPL patterns may be limited in their interpretation of biological activity for noisy regions and that the overall acoustic signature that we find in more pristine estuaries is lost in Charleston Harbor.

## 1. Introduction

Marine ecosystems face many stressors (*e*.*g*., noise pollution, overfishing, contaminants, and climate change) that can be difficult to quantify due to limitations in access, visibility, and dynamic conditions. Since sound travels efficiently in water, monitoring biological sound and anthropogenic noise allows for “eavesdropping” on habitats through passive acoustic monitoring [1]. Acoustic signals can be important for reproduction of soniferous (*i*.*e*., sound-producing) fish species [2–4], larval settlement [5–7], and predator avoidance [8–10]. Thus, biological sound provides information about species and ecosystem health as well as their response and resilience to anthropogenic and environmental stressors. Soundscape ecology uses this knowledge to provide a long-term, noninvasive approach to track animal behavior, habitat quality, and community structure over time and space at fine-scale resolutions [11].

Rapid development in South Carolina (SC), United States (US) is changing coastal ecosystems and increasing the pressure of anthropogenic stressors on marine ecosystems [12]. Charleston County’s population is growing at three times the national average with a 17.6% increase from 2010–2019 [13]. This growth leads to a higher magnitude of commercial and recreational vessel operations and thus, increased noise pollution [14]. Anthropogenic noise disrupts acoustic signals and has been an increasing issue in marine ecosystems over the last century [15–17]. Long-term monitoring of how coastal organisms and ecosystems are changing in response to anthropogenic noise is lacking [12]. Estuaries like Charleston Harbor provide important nursery habitats along the SC coastline and are highly productive ecosystems, supporting an abundance of marine life including soniferous invertebrates, fish, reptiles, and mammals [18–20]. While acoustic methods have been used to locate fish spawning [21, 22], no full soundscape studies have been completed in Charleston Harbor.

Soundscape studies in Chechessee Creek and the May River estuary, further south along the SC coastline, have identified snapping shrimp (*Alpheus spp*. and *Synalpheus spp*.); fish belonging to the families Sciaenidae [*i*.*e*., Atlantic croaker (*Micropogonias undulatus*), silver perch (*Bairdiella chrysoura*), black drum (*Pogonias cromis*), spotted seatrout (*Cynoscion nebulosus*), and red drum (*Sciaenops ocellatus*)] and Batrachoididae [*i*.*e*., oyster toadfish (*Opsanus tau*)]; and common bottlenose dolphins (*Tursiops truncatus*; [4, 23]). Snapping shrimp are the primary biological sound producers in subtropical habitats [23–26]. They are abundant, crevice-dwelling, benthic organisms that can be found and heard most frequently in rocky substrates including oyster reefs [23, 26–30]. Their short (<0.1 s), loud, and broadband frequency (1 Hz—200 kHz) snaps are used in communication, foraging, and territorial behavior [24, 25, 31–33]. Sciaenids and Batrachoidids create low frequency (50–1200 Hz) species-specific calls that are used to attract mates during courtship [34–39]. This allows for acoustic monitoring and the determination of reproductive potential within a spawning season [2, 36–39]. Common bottlenose dolphins are apex predators in SC estuaries in which Sciaenids and Batrachoidids comprise a substantial percentage of their diet [9, 40–42]. Common bottlenose dolphins use three main vocalization types, which include whistles and burst pulse sounds used for communication, and echolocation used in foraging and navigation [43–45].

Research shows that anthropogenic noise can result in a variety of consequences for marine organisms including masking of communication signals, increased stress levels, hearing threshold shifts, and damage to auditory structures [46–49]. Masking of auditory signals by anthropogenic noise can result in missed foraging opportunities, failure to respond to the presence of a predator or vessel, or loss of social cues for group cohesion, which may affect the formation of fish spawning aggregations [50–54]. Behavioral stress responses to anthropogenic noise for fish and marine mammal species can include the interruption of foraging and social activities through triggering of a flight response [48, 55, 56] or through the avoidance of key habitat to evade excessive noise [57]. Intense or persistent anthropogenic noise has the ability to damage auditory structures in both fishes and marine mammals [58, 59].

The goal of this study was to evaluate soundscape patterns in Charleston Harbor following comparable methodologies used in less developed estuaries of SC including Chechessee Creek [60] and the May River estuary [4, 23, 61]. Understanding soundscape patterns in Charleston Harbor will provide insights into the behavior and interactions of species within an urbanized estuary and provide a non-invasive technique with high temporal resolution to monitor organismal response to anthropogenic stressors like noise pollution. This work can then provide a model approach for managers to compare soundscape endpoints among estuaries of varying levels of anthropogenic stressors.

## 2. Materials and methods

### 2.1 Study area

**Fig 1:**
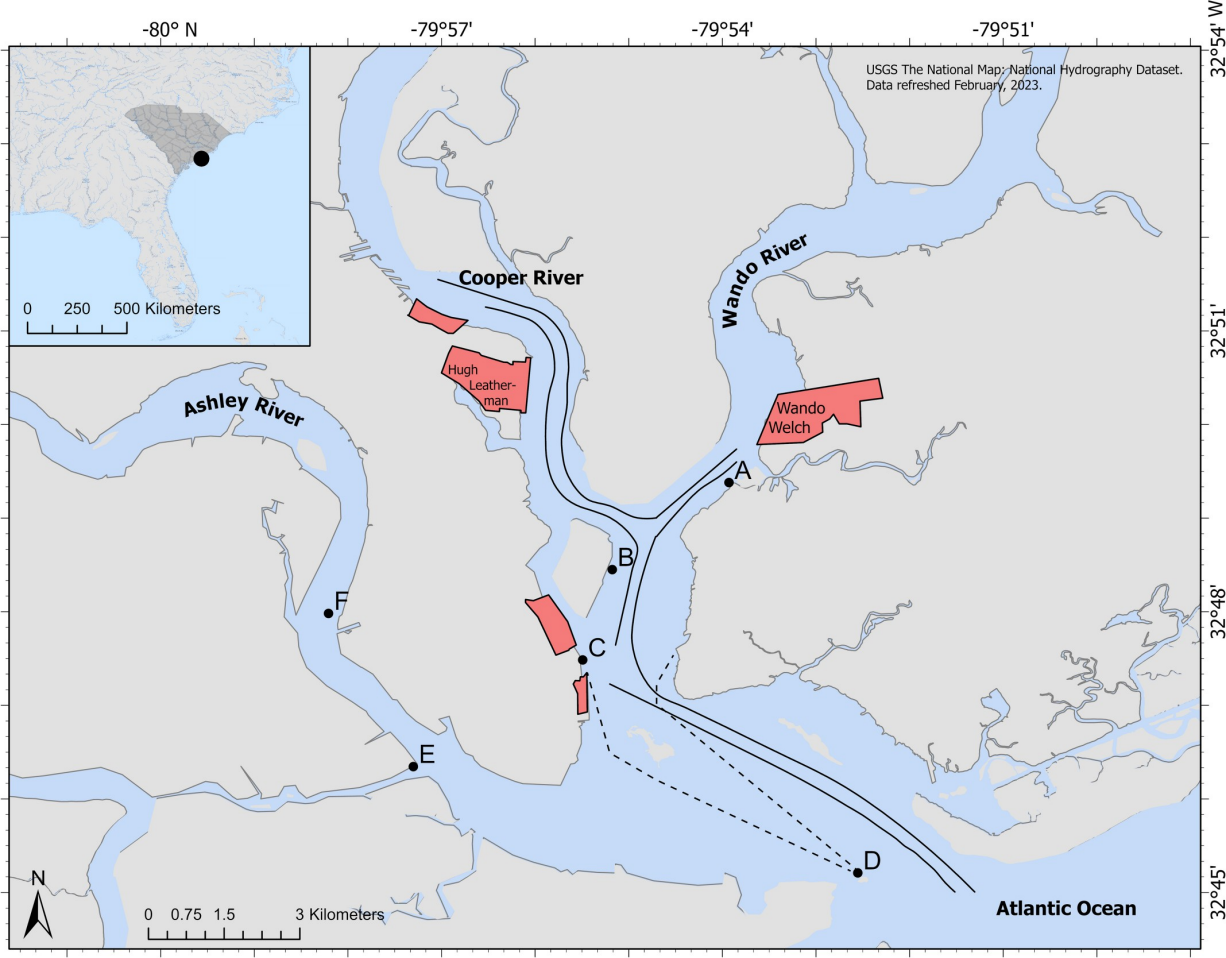
Passive acoustic monitoring station map. Charleston Harbor, South Carolina (SC) study area with passive acoustic recorder stations that were deployed from December 2017—June 2019. Stations include (A) The Wando River (B) Drum Island (C) the SC Aquarium (D) Fort Sumter (E) the Ashley River, and (F) the Citadel. Black lines show the shipping channel. Red shapes show shipping terminals. Black dotted lines show the ferry path to Fort Sumter from the SC Aquarium and Patriot’s Point. The base map is the USGS Hydro Cache Base Map.

Charleston Harbor (32°49′7″ N, 79°55′40″W) is a partially mixed estuary, where the Ashley, Cooper, and Wando Rivers flow into the Atlantic Ocean through the harbor (Fig 1; [62]). This estuary is part of the central SC coastline and positioned within the South Atlantic Bight. Charleston Harbor is lined with salt marshes dominated by eastern oyster reefs (*Crassostrea virginica*), and smooth cordgrass (*Spartina alterniflora*), and this estuary experiences semi-diurnal tides with a tidal range of 1.8 m [63]. Both commercial and naval ports are within Charleston Harbor, serving as the busiest and fastest growing harbor in SC [64]. Charleston Harbor covers a large area, approximately 36 km^2^. While typical depth ranges from 3–9 m, navigation channels are maintained at an average depth of 13.7 m and the harbor entrance is maintained at 14.3 m [65].

Six passive acoustic monitoring stations were deployed in Charleston Harbor from December 2017 to June 2019 (Fig 1). Station A, called the “Wando River”, was located across from Daniel Island and near the Hobcaw Yacht Club and the SC Port Authority’s Wando Welch Terminal, with an average depth of 2.8 m, average salinity of 21.2 ± 3.1 ppt, and average pH of 8.1 ± 0.4. Station B, “Drum Island”, was located at the mouth of the Cooper River underneath the Ravenel Bridge, with an average depth of 3.3 m, average salinity of 23.1 ± 2.3 ppt, and average pH of 8.2 ± 0.4. Station C, “SC Aquarium”, was located at the SC Aquarium dock near the junction where the Cooper and Wando Rivers flow into Charleston Harbor, with an average depth of 2.9 m, average salinity of 23.1 ± 3.5 ppt, and average pH of 8.2 ± 0.4. Station D, “Fort Sumter”, was located at the mouth of the harbor at Fort Sumter National Monument, with an average depth of 4.2 m, average salinity of 27.2 ± 3.9 ppt, and average pH of 8.2 ± 0.5. Station E, “Ashley River”, was located near the mouth of the Ashley River at the Wappoo Cut and across from the Charleston Yacht Club, with an average depth of 3.0 m, average salinity of 24.4 ± 2.6 ppt, and average pH of 8.0 ± 0.3. Station F, “Citadel”, was located at a private dock further in the Ashley River and on the downtown side near the Citadel, with an average depth of 2.8 m, average salinity of 22.8 ± 2.8 ppt, and average pH of 7.8 ± 0.3. Depth over the study period ranged from 0.5–5.9 m depending on the station and tidal cycle, and salinity ranged from 18.3 to 31.5 ppt. The Wando River, Drum Island, SC Aquarium, and Fort Sumter stations lie adjacent to the shipping channel (average depth of 13.7 m). Permits for recorder deployment were not required at The Wando River (A), Drum Island (B), SC Aquarium (C), Ashley River (E), or Citadel (F) locations because permission was granted by private businesses, citizens, or the SC Ports Authority. A permit for the Fort Sumter (D) location was granted by the National Park Service.

### 2.2 Environmental and acoustic data collection

Passive acoustic recorders (DSG-ST, Loggerhead Instruments; gain set to 33 dB) with HTI-96-Min hydrophones (High Tech, Inc; hydrophone sensitivity of -201 dB re: 1V/μPa) were tested, mounted in custom built frames (Mooring Systems), painted with antifouling paint (Trilux 33), and then deployed for 90 days at a time with a sample rate of 96 kHz and functional listening range of 0.001–48 kHz following methods previously described [4, 23, 60, 61]. During deployments, water quality measurements including salinity, temperature, and pH were measured at each station using a YSI 556 Handheld Multiparameter Instrument (YSI Inc./Xylem Inc., Yellow Springs, OH). PVC housings were attached to the instrument frames with zip ties containing a water temperature logger (HOBO Water Temperature Pro v2 U22-001, Onset Computer Corporation) and water level logger (HOBO 100-Foot Depth Water Level Data Logger U20-001-02-Ti) that recorded temperature and depth every 20 minutes. Acoustic recordings occurred for two minutes every twenty minutes. Each recorder was deployed from December 11, 2017 to June 3, 2019 with week-long gaps in data collection every 90 days during servicing. Before each deployment, tones were played at multiple frequencies (*i*.*e*., 100, 200, 400, 800, 1600, 3200, 6400, and 8000 Hz) and then root mean square (rms) sound pressure levels (SPLs) were calculated for each frequency to test the functionality of the recording system and sound levels. After deployment, acoustic recordings were downloaded and batch converted from DSG files into.wav files using DSG2wav© software (Loggerhead Instruments).

In shallow water environments (<10 m) like Charleston Harbor, there is considerable variation in maximum detection range of soniferous fish, bottlenose dolphins, and anthropogenic noise sources because sound propagation is complex and varies by habitat type and water depth [66–68]. A recent study in similar habitat used playback studies of spotted seatrout calls to determine that the detection range for soniferous fish was less than 281 m [68]. Another study using similar recorders (DSG Ocean recorders with a hydrophone sensitivity of −186 dBV/μPa) determined a detection range of 200–300 m for bottlenose dolphin whistles using a cylindrical spreading model on the West Florida Shelf [69]. A Cardigan Bay, Wales study in deeper water (17–22 m) found the detection range of bottlenose dolphin vocalizations at approximately 1.14 km [70]. A study in the Hudson River shipping lane detected ships from a range of 2–7 km [71]. Since our study sites ranged from 1.8–8.0 km apart, it is unlikely that fish calls or bottlenose dolphin vocalizations were simultaneously recorded at multiple stations; it is possible that ship noise was detected at multiple stations along the shipping channel.

### 2.3 Acoustic review

For manual analysis, observers reviewed two-minute recordings every hour from the original duty cycle (*i*.*e*., two-minute recordings every 20 minutes). This approach was completed for the entire study resulting in 70,493.wav files manually reviewed. Adobe Audition CS5.5 software (Adobe Systems) was used to manually identify fish calling, dolphin vocalizations, physical sounds (*i*.*e*., waves, water flow, and rain), and anthropogenic noise (*i*.*e*., recreational boats, ferries, and commercial vessels). Spectrograms were reviewed and sound signals of each species were identified by comparison to previously published spectrograms [23, 72–75]. Fish calling was scored on a scale of 0 to 3 (*i*.*e*., 0 = no calls; 1 = 1 call; 2 = multiple calls; and 3 = overlapping calls or chorusing) for all species in each 2-minute.wav file as previously described [4, 37]. Dolphin vocalizations were characterized as echolocation, burst pulses, and whistles and counted individually for each 2-minute.wav file and then summed per file for a total vocalization count [23, 60, 76]. Infrequent baleen whale vocalizations were identified as North Atlantic right whale (*Eubalaena glacialis*) tonal calls [75] and scored as present (score of 1) or absent (score of 0). Physical sounds and anthropogenic noise were separately scored as present (score of 1) or absent (score of 0; [23, 60, 61]).

Custom MATLAB version R2017b (MathWorks, Inc., Natick, MA, USA) scripts were used to calculate average root mean square (rms) sound pressure levels (SPLs) of low (50–1200 Hz) and high (7000–40000 Hz) frequency ranges for each 2-minute.wav file (*i*.*e*., every 20 min). These ranges were chosen based on previous studies that analyzed frequencies of soniferous species common to SC estuaries including snapping shrimp (50–40,000 Hz), oyster toadfish (190–200 Hz), silver perch (1000–1280 Hz), black drum (70–90 Hz), spotted seatrout (200–270 Hz), and red drum (120–160 Hz; [4, 74]). Low frequency SPLs primarily represent fish calling but also include the lower frequency portion of snapping shrimp snaps, bottlenose dolphin vocalizations (*i*.*e*., burst pulses), physical sounds, and anthropogenic noise. High frequency SPLs primarily represent snapping shrimp snaps but also include upper frequency dolphin vocalizations (*i*.*e*., burst pulses, whistles, and echolocation), physical sounds, and anthropogenic noise. Using the manually reviewed dataset, files containing anthropogenic noise were then removed to create a subset of low and high frequency SPLs for further analysis. This procedure ensured that the observed patterns in SPLs were primarily of biological nature. High frequency SPLs have been used to represent snapping shrimp acoustic activity in other studies since previous work has shown strong correlations between these parameters [77–79]. In this study, the high frequency SPL subset without anthropogenic noise was used to represent snapping shrimp acoustic activity to eliminate the contribution of anthropogenic noise in the similar frequency range.

### 2.4 Statistics

Random forest modeling was used to investigate the influence of various factors on sound pressure levels, fish calling, dolphin vocalizations, and anthropogenic noise detections. Random forest modeling is an ensemble machine learning technique that conducts bootstrap training and testing using decision trees on two-thirds of a dataset [80]. It then provides a majority voting result (for classification models) or an average result (for regression models) that it compares to the other third of the dataset to provide an “out of bag” estimate of error rate (for classification models) or a percent of explained variance (for regression models; [80]). Out of bag estimates of error rates were subtracted from 100% to calculate the prediction accuracy of each model. This approach allows testing of non-parametric data that is not independent, not sampled randomly (*i*.*e*., collected at 20-minute intervals), includes gaps in data collection, and has a mix of continuous and categorical variables that would violate assumptions of other statistical approaches [80].

The influences of recorder station, month or water temperature, lunar phase, day/night cycles, and tidal phase on low and high SPLs (with and without anthropogenic noise) were tested with random forest modeling using the “randomForest” package in R version 4.1.1 [80]. In addition, this type of modeling investigated the effects of recorder station, month or water temperature, lunar phase, day/night cycles, or the presence of vessel noise on fish calling by species and total dolphin vocalizations. Furthermore, the influences of month or water temperature, recorder station, weekday, and day/night cycles on anthropogenic noise detections were tested. Lunar and tidal phases were categorized as previously described [4, 23]. Day and night were classified by daily sunrise and sunset times. Models were run with both water temperature and month separately to remove collinearity; the model with the most explained variation for regression models (continuous response variables: SPLs, bottlenose dolphin vocalizations) or the highest prediction accuracy for classification models (categorical response variables: scores of fish calling intensity and anthropogenic noise detection) were reported. After testing and comparing model accuracy and error rates, the default values were used in each random forest model with the seed set to 42 and p-value set to 0.01 [61, 80, 81]. The “Boruta” package in R version 4.1.1 was used to calculate mean importance scores (Z-scores) and rank variables [82, 83]. The Dunnett-Tukey-Kramer multiple comparison test was then applied post-hoc with a 95% confidence interval to determine significant differences within ranked variables [84].

To decrease the possibility of collinearity, fish calling and dolphin vocalization models were then limited to each species’ calling season and circadian pattern to confirm any significant relationships between anthropogenic noise detections and biological sounds. Targeted models were selected based on the patterns displayed in this dataset and are as follows: black drum (March–April, 15:00–0:00), oyster toadfish (March–June, all hours), silver perch (March–June, 15:00–5:00), spotted seatrout (March–September, 14:00–2:00), red drum (August–October, 13:00–20:00), and bottlenose dolphins (November–February, all hours).

In order to quantify biological and anthropogenic sound contributions to SPLs, random forest models were created with low frequency SPLs as the dependent variable and each fish species calling intensity, the number of bottlenose dolphin vocalizations, and presence of anthropogenic noise as independent variables. Separate random forest models were designed for each season and diurnal category (day or night). Seasons were applied with the astronomical start dates for the Northern Hemisphere: spring begins March 20, summer begins June 21, fall begins September 22, and winter begins December 21. This statistical model was repeated for high frequency SPLs with bottlenose dolphins and anthropogenic noise detection as the independent variables. The “Boruta” package in R was then used to rank the contribution of sound producers to SPLs. The Dunnett-Tukey-Kramer multiple comparison test was applied post-hoc to determine whether the relationship was positive or negative. Negative relationships (*e*.*g*., lower SPLs when calling occurred) were removed because this pattern indicated that calling did not contribute to the low or high frequency SPLs.

## 3. Results

### 3.1 Patterns of sound pressure levels

**Fig 2:**
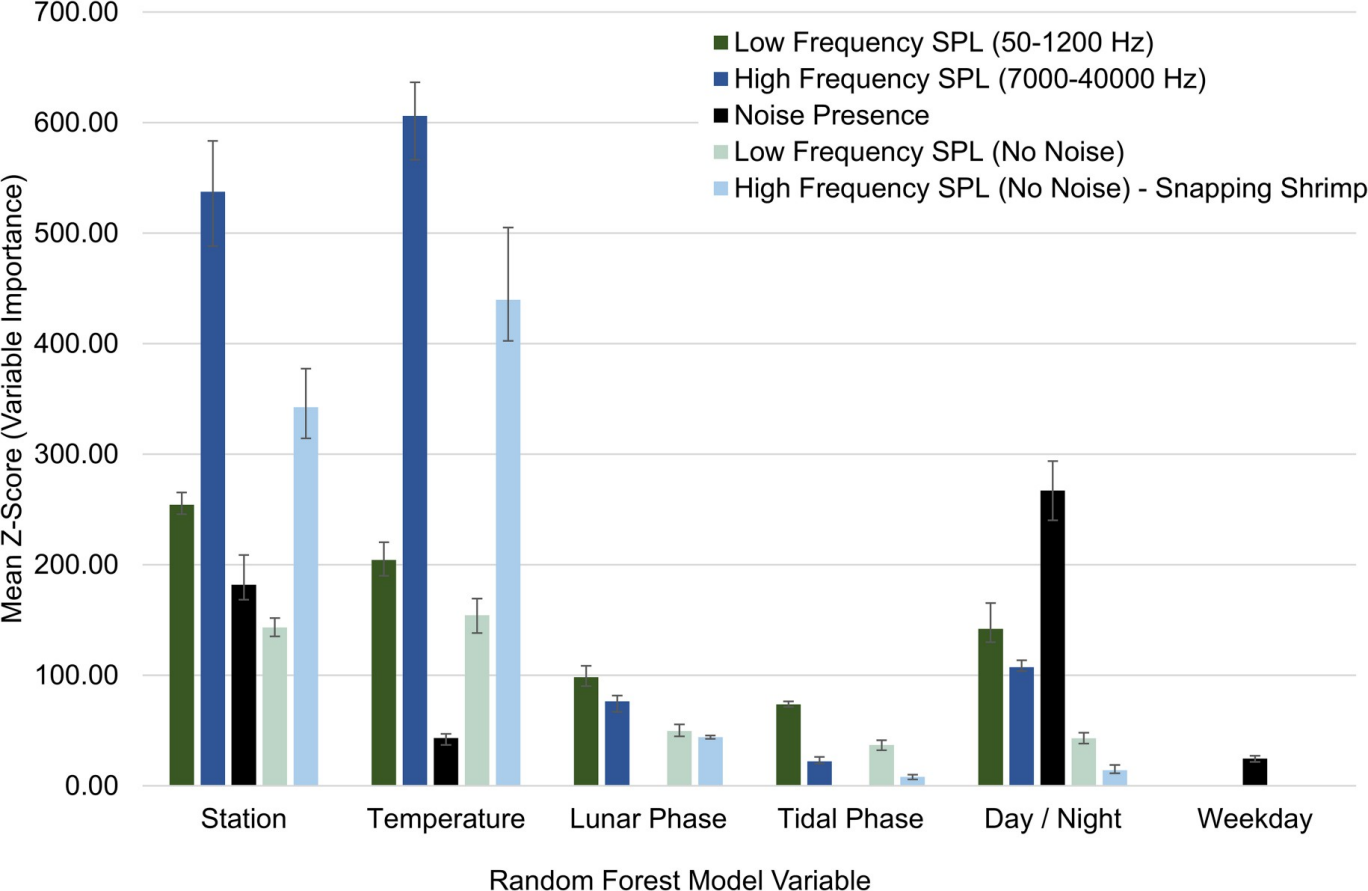
Boruta, random forest model variable significance and ranking for sound pressure levels and anthropogenic noise detections. Influence of spatial and temporal variables on low and high frequency sound pressure levels (SPLs), anthropogenic noise presence, and low (indicative of all fish calling) and high (indicative of snapping shrimp acoustic activity) frequency SPLs excluding noise files. The low and high frequency SPLs with noise used data every 20 minutes. Anthropogenic noise presence as well as low and high frequency SPLs excluding noise used data that were manually analyzed every hour. Bars represent the significance (mean Z-score) of each variable from the Boruta wrapper algorithm based on a random forest model. Z-score is not a statistical value, it is a comparison of data to a randomized version of the data. Error bars represent the minimum and maximum Z-score. All the factors were significant at p < 0.01. Post hoc differences can be seen in Table 1.

**Fig 3:**
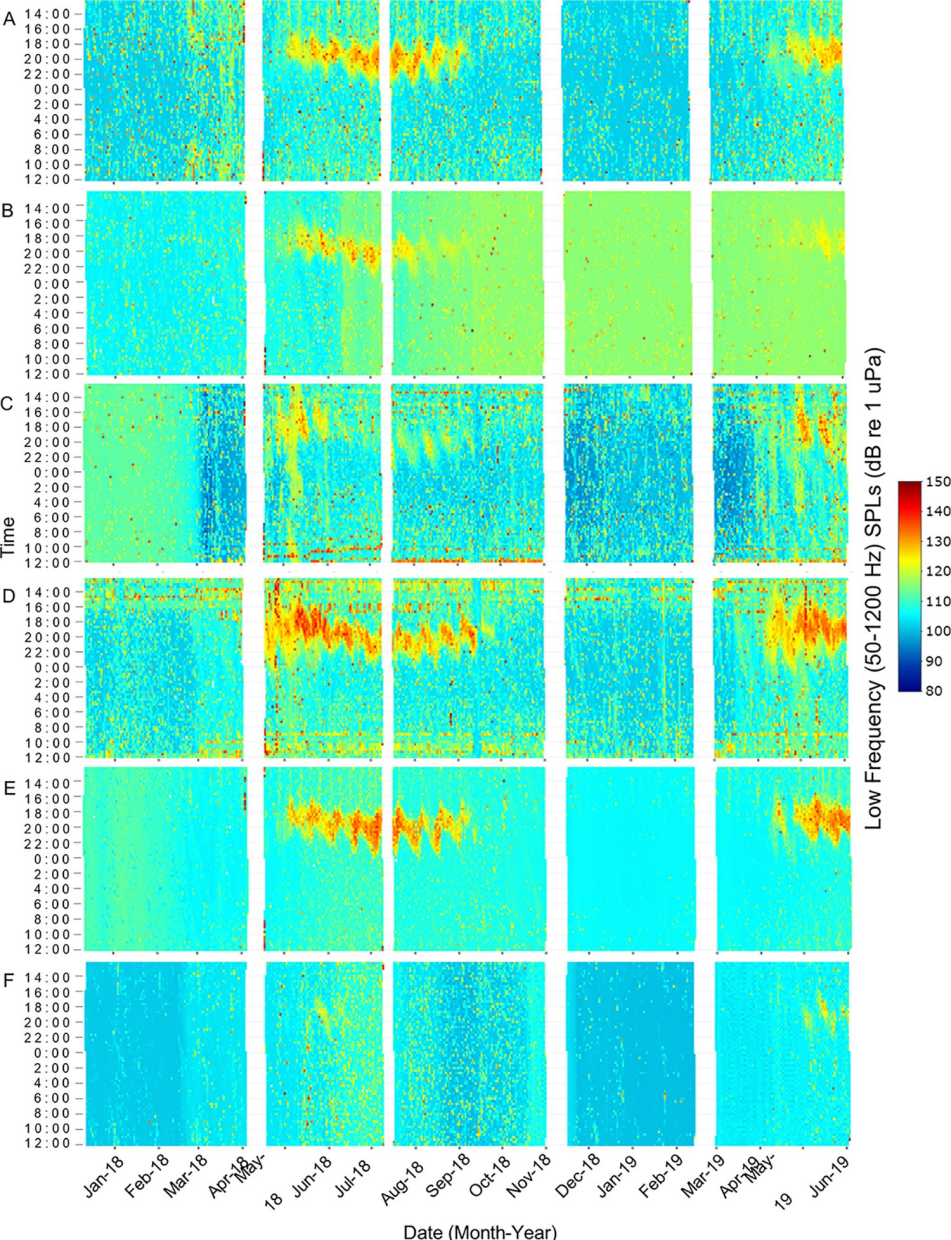
Low frequency, sound pressure level heat map. Spatial and temporal patterns of low frequency received sound pressure levels (50–1200 Hz) at (A) Wando River, (B) Drum Island, (C) SC Aquarium, (D) Fort Sumter, (E) Ashley River, and (F) Citadel stations. Recordings were taken every 20 minutes. Time is shown between noon and noon the next day to display the overnight fish chorusing signatures. White spaces represent gaps in the data due to maintenance of equipment between deployments.

**Fig 4:**
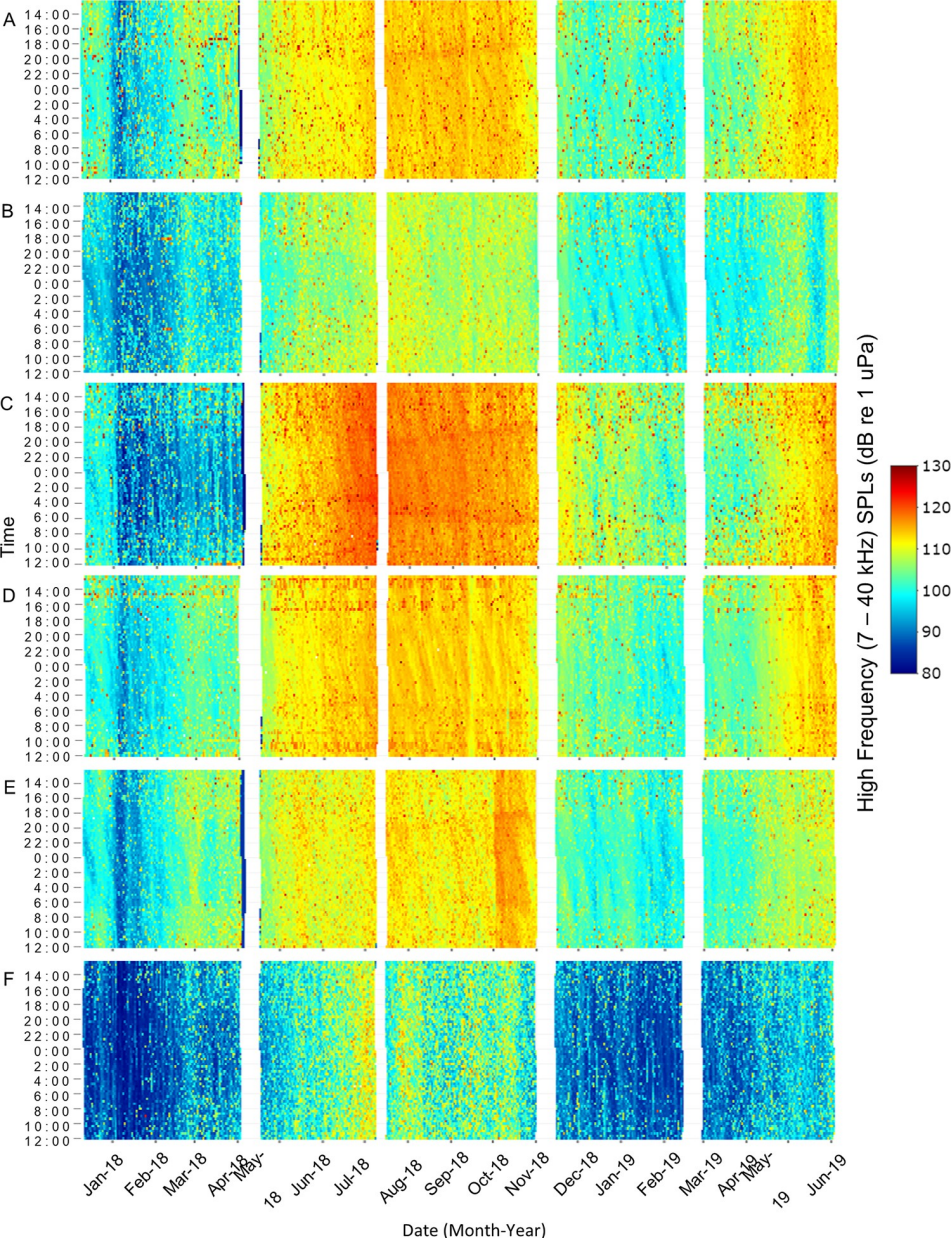
High frequency, sound pressure level heat map. Spatial and temporal patterns of high frequency received sound pressure levels (7–40 kHz) at (A) Wando River, (B) Drum Island, (C) SC Aquarium, (D) Fort Sumter, (E) Ashley River, and (F) Citadel stations. Recordings were taken every 20 minutes. Time is shown between noon and noon the next day. White spaces represent gaps in the data due to maintenance of equipment between deployments.

Low frequency received SPLs (50–1200 Hz) primarily included fish calling and anthropogenic noise as well as the lower bandwidth of snapping shrimp snaps, bottlenose dolphin vocalizations, and physical sounds. High frequency received SPLs (7000–40000 Hz) primarily included snapping shrimp snaps as well as the upper bandwidth of bottlenose dolphin vocalizations, anthropogenic noise, and physical sounds. Both SPL ranges exhibited spatial and temporal differences that were influenced by water temperature, day/night, lunar phase, and tidal phase (Fig 2, p < 0.01). These variables explained 33.7% and 76.1% of the variance in low and high frequency SPLs, respectively (Table 1). Station and water temperature were the most important variables in both SPL models (Fig 2). Low frequency SPLs were highest at Drum Island, while high frequency SPLs were highest at the SC Aquarium (Table 1, Figs 3 and 4, p < 0.001). Overall, SPLs were highest in the summer months, with peak low frequency SPLs observed in May and June (Table 1, Fig 3, p < 0.001) and peak high frequency SPLs observed in July (Table 1, Fig 4, p < 0.001). Low frequency SPLs decreased towards the winter months and were equally low from November to February, while high frequency SPLs experienced a slower decrease into the winter months with the lowest SPLs observed in January coinciding with the lowest temperatures (S1 Table, Figs 3 and 4, p < 0.001). The new moon was the quietest lunar phase in the estuary (S1 Table, p < 0.001). Low frequency SPLs were greatest during the full moon and quietest during the new moon (Table 1, p < 0.001). High frequency SPLs, however, were greatest during the first quarter and lowest during the third quarter and new moon phases (Table 1, p < 0.001). Daytime was louder than nighttime for both low and high frequency SPLs (Table 1, p < 0.001). Low frequency SPLs were greatest at the high tide and lowest at low tide (Table 1, p < 0.02). Post-hoc testing did not display tidal differences in high frequency SPLs, but it was confirmed in the random forest model. Tidal influences were observed as diagonal patterns in the high frequency SPL heat maps that matched the high tide (Table 1, Fig 4, S1 Fig).

**Table 1:** Top ranked variable in each random forest model for low and high frequency root mean square (rms) sound pressure levels (SPLs), anthropogenic noise presence, and low (indicative of all fish calling) and high (indicative of snapping shrimp acoustic activity) frequency SPLs excluding noise files. Blank cells indicate that variable was not included in the associated model. Fig 2 shows the relative rank of each factor.

	Station df = 5	Month / Temperature df = 11 / df = 1	Lunar phase df = 3	Tidal phase df = 3	Day / night df = 1	Weekday df = 6	% variation explained / prediction accuracy*
**Low Frequency SPL (50–1200 Hz)**	Drum Island (B)	May, June	full moon	high	day		33.71%
**High Frequency SPL (7,000–40,000 Hz)**	SC Aquarium (C)	July	first quarter	no differences	day		76.10%
**Noise Detection**	SC Aquarium (C) (shipping channel stations > Ashley River stations)	June			day	Saturday	72.48%*
**Low SPL—No Noise**	Drum Island (B)	May, June, July	full moon	high	night		45.47%
**High SPL—No Noise**	Wando River (A)	July	first quarter	high	night		82.88%

**Fig 5:**
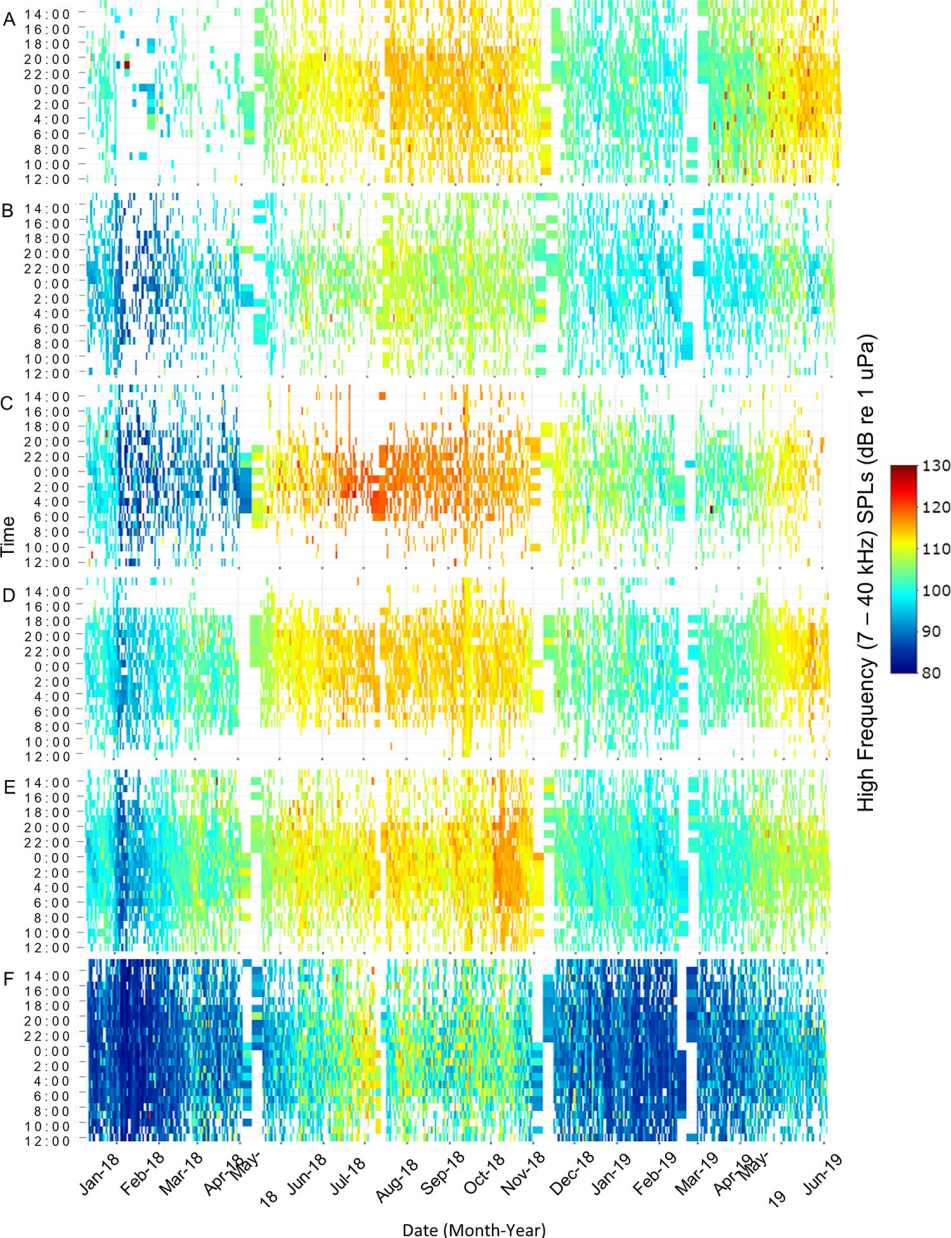
High frequency, sound pressure level heat map—noise excluded. Spatial and temporal patterns of high frequency received sound pressure levels (7–40 kHz) with all detections of noise excluded at (A) Wando River, (B) Drum Island, (C) SC Aquarium, (D) Fort Sumter, (E) Ashley River, and (F) Citadel stations. Two minutes of acoustic data from each hour were manually reviewed. Time is shown between noon and noon the next day. White spaces represent gaps in the data due to maintenance of equipment between deployments or removal of a recording from the dataset due to the detection of anthropogenic noise.

When files with anthropogenic noise were removed from the dataset, SPLs revealed different spatial and temporal biological patterns that were originally masked by anthropogenic noise (Table 1). Temporally, low and high SPLs with anthropogenic noise included in the dataset were louder during the day (Table 1, Figs 3 and 4). When anthropogenic noise files were removed, low and high SPLs were louder at night (Table 1, Fig 5, low frequency SPL heat maps with noise removed not shown, p < 0.02).

## 3.2 Identified sounds and anthropogenic noise

**Fig 6:**
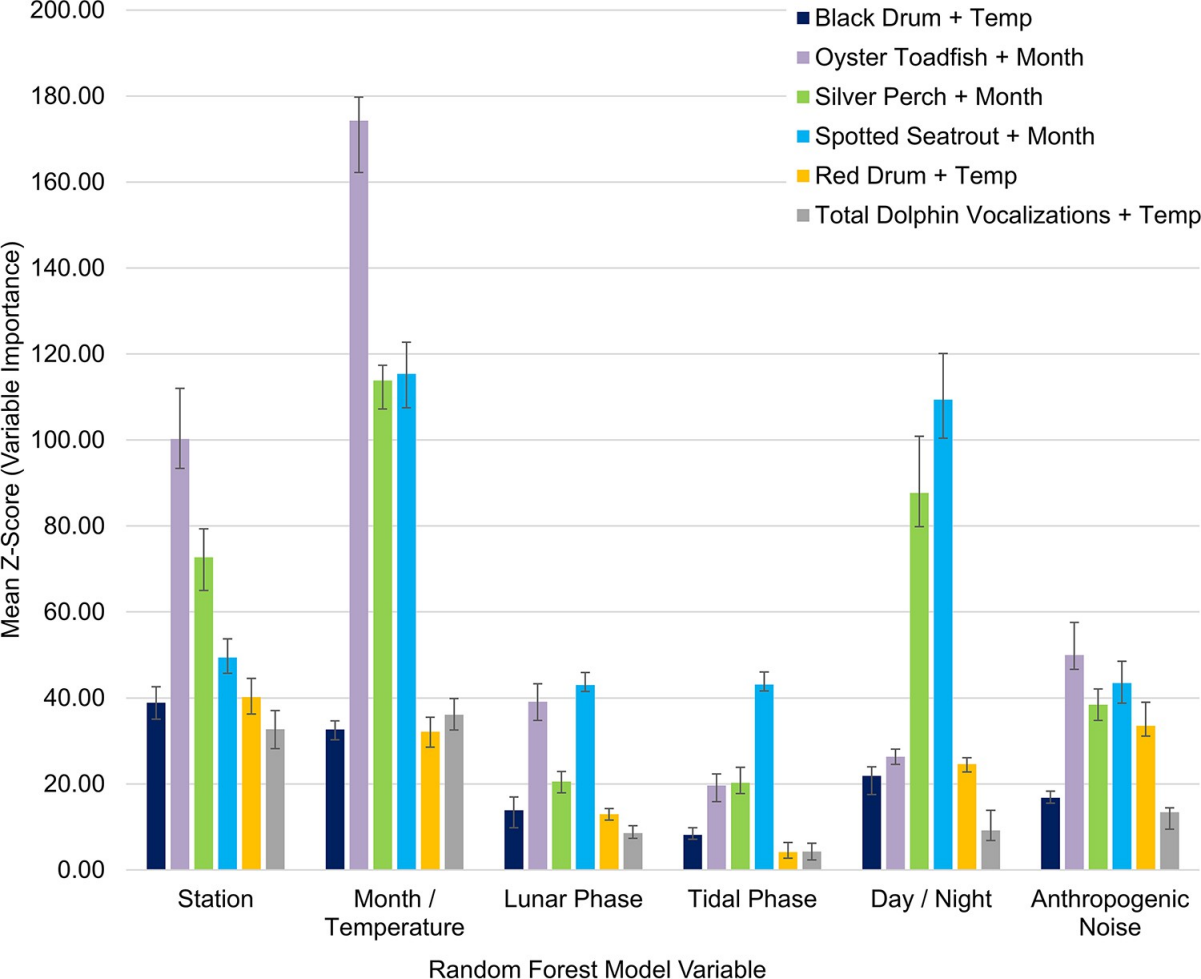
Boruta, random forest model variable significance and ranking for biological sound producers. Influence of spatial, temporal, and anthropogenic noise variables on calling and vocalizing for black drum, oyster toadfish, silver perch, spotted seatrout, red drum, and bottle dolphins. These models used data that were manually analyzed every hour. Bars represent the significance (mean Z-score) of each variable from the Boruta wrapper algorithm based on a random forest model. Z-score is not a statistical value, it is a comparison of data to a randomized version of the data. Error bars represent the minimum and maximum Z-score. All the factors were significant at p < 0.01. Post hoc differences can be seen in Table 3.

**Fig 7:**
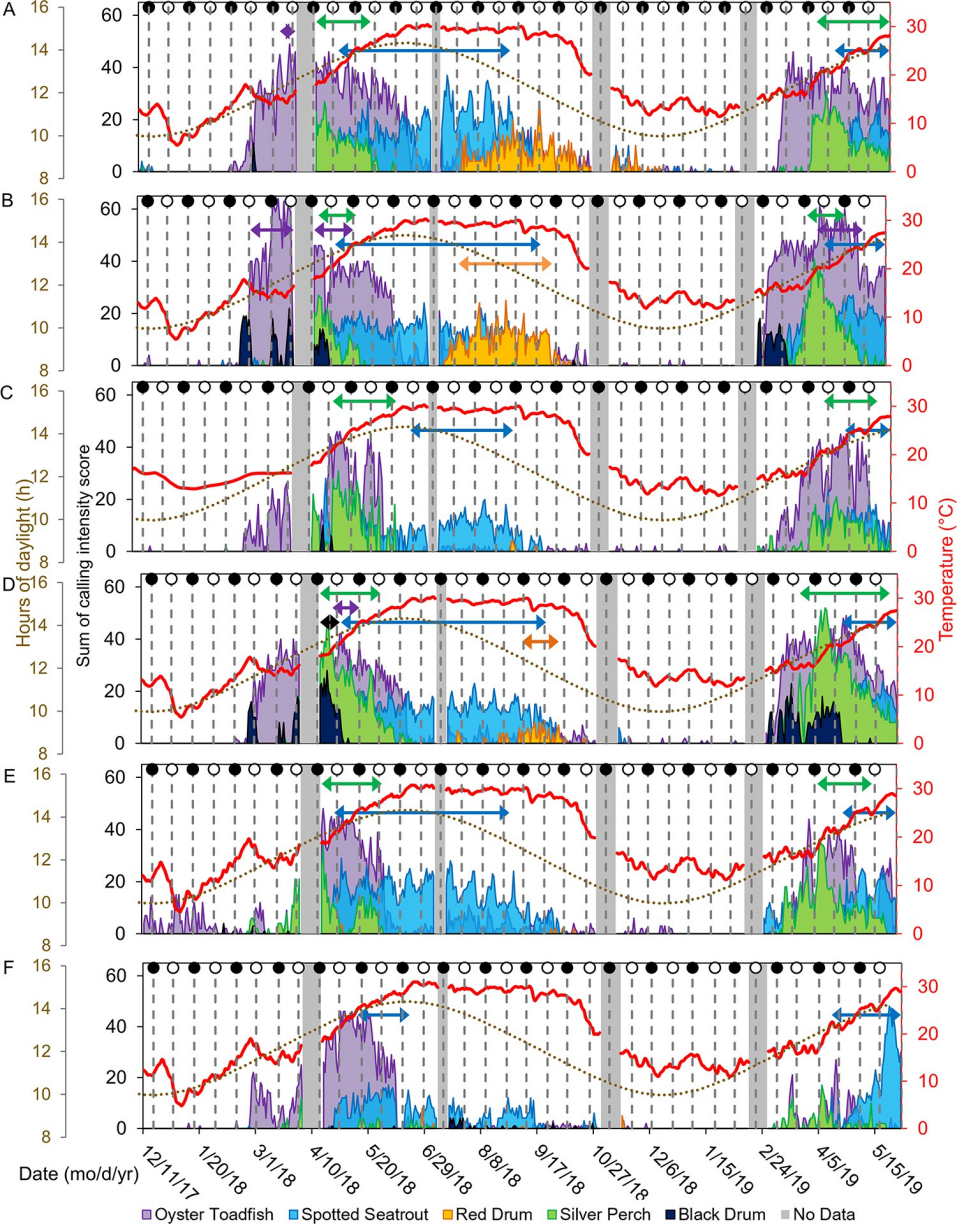
Time series of fish calling. Seasonal and spatial patterns of fish sound production in Charleston Harbor. Sums of calling intensity scores are shown from noon to noon the next day at (A) Wando River, (B) Drum Island, (C) SC Aquarium, (D) Fort Sumter, (E) Ashley River, and (F) Citadel stations. Also shown are hours of daylight (brown dotted line), average daily water temperature from HOBO loggers (red line), and new (dark circles) and full (white circles) moon phases. Grey bars represent gaps in the data due to maintenance of equipment between deployments. Colored arrows show chorusing date range for each species.

**Fig 8:**
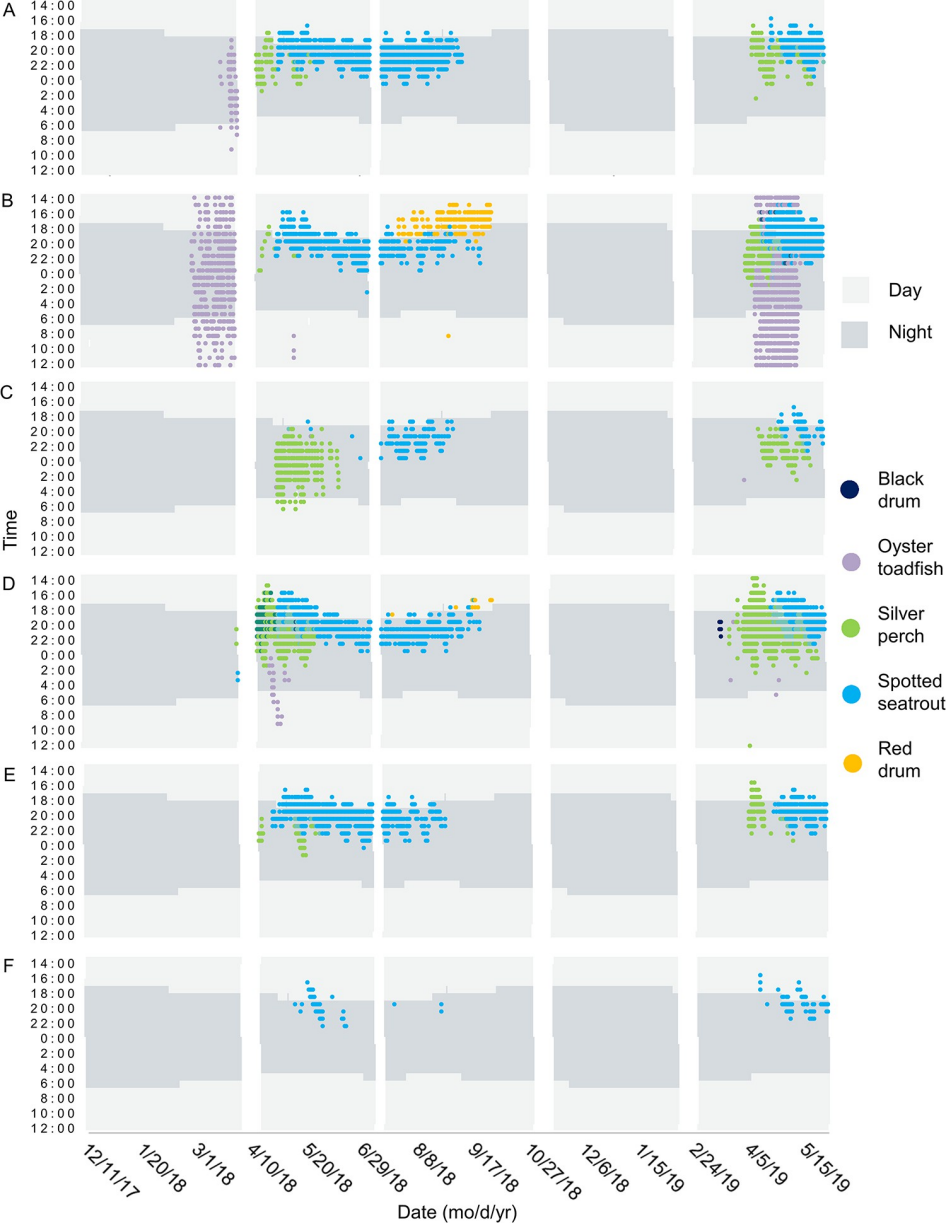
Heat map of fish chorusing with day/night background. Spatial and temporal patterns of fish chorusing in Charleston Harbor. Time is shown between noon and noon the next day at (A) Wando River, (B) Drum Island, (C) SC Aquarium, (D) Fort Sumter, (E) Ashley River, and (F) Citadel stations. Background color represents whether the time is during the day or night. White spaces represent gaps in the data due to maintenance of equipment between deployments. Blended colors indicate an overlap in species calling.

**Fig 9:**
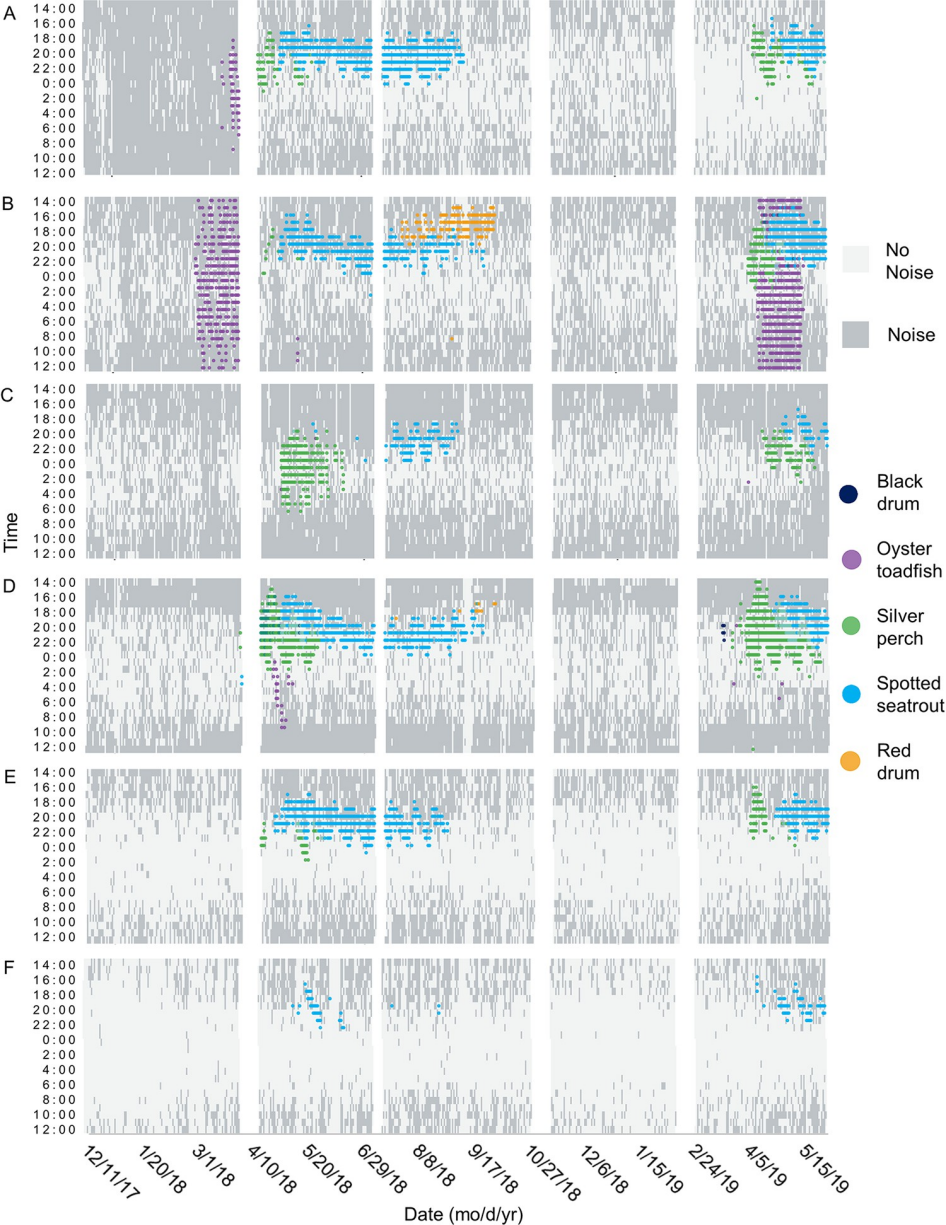
Heat map of fish chorusing with noise presence/absence background. Spatial and temporal patterns of fish chorusing in Charleston Harbor. Time is shown between noon and noon the next day at (A) Wando River, (B) Drum Island, (C) SC Aquarium, (D) Fort Sumter, (E) Ashley River, and (F) Citadel stations. Background color represents whether anthropogenic noise was present or absent. White spaces represent gaps in the data due to maintenance of equipment between deployments. Blended colors indicate an overlap in species calling.

**Fig 10:**
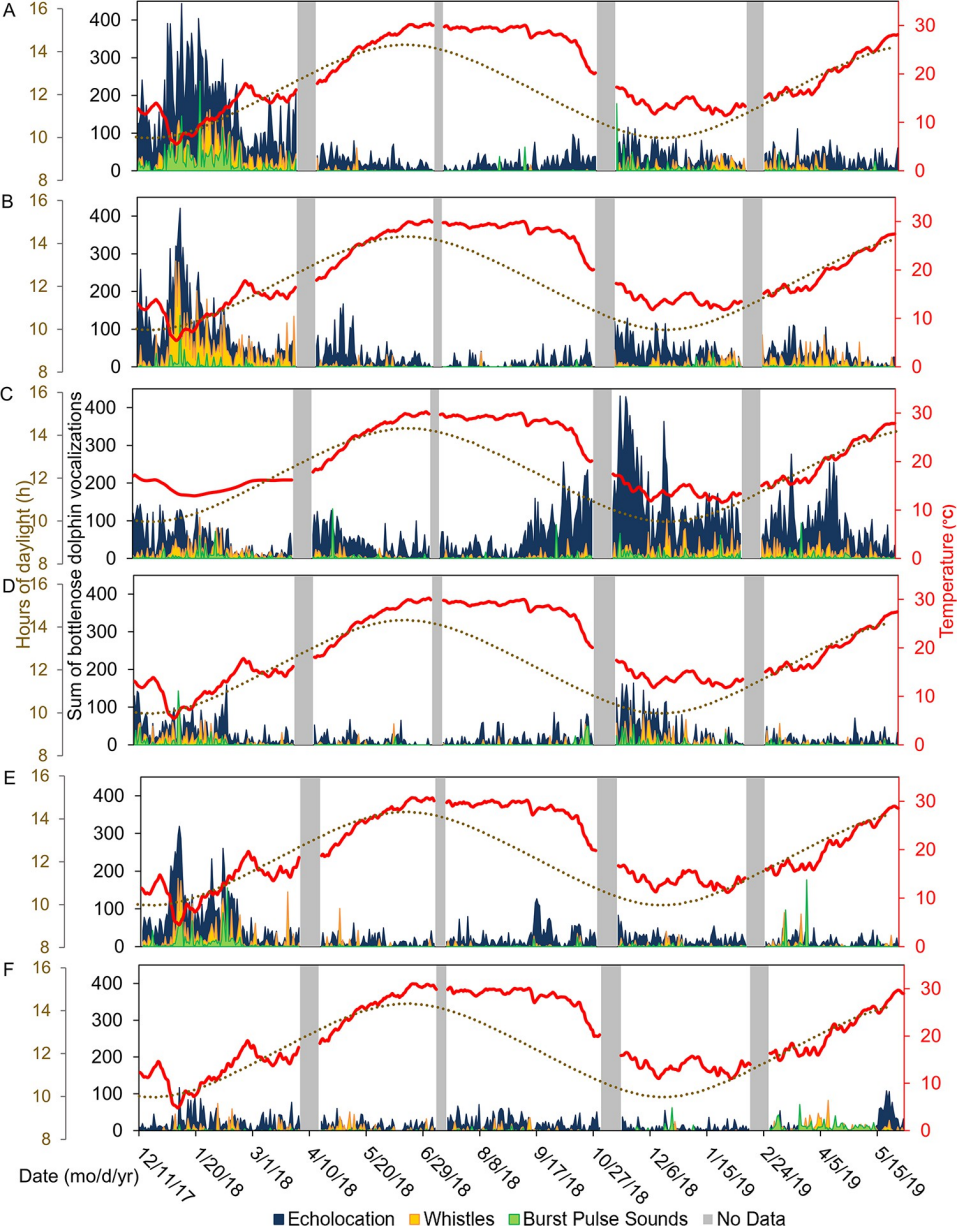
Time series of bottlenose dolphin vocalizations. Seasonal and spatial patterns of bottlenose dolphin vocalizations in Charleston Harbor, SC. Sums of vocalizations are shown from noon to noon the next day at (A) Wando River, (B) Drum Island, (C) SC Aquarium, (D) Fort Sumter, (E) Ashley River, and (F) Citadel stations. Also shown are hours of daylight (brown dotted line) and average daily water temperature (red line). Grey bars represent gaps in the data due to maintenance of equipment between deployments.

**Fig 11:**
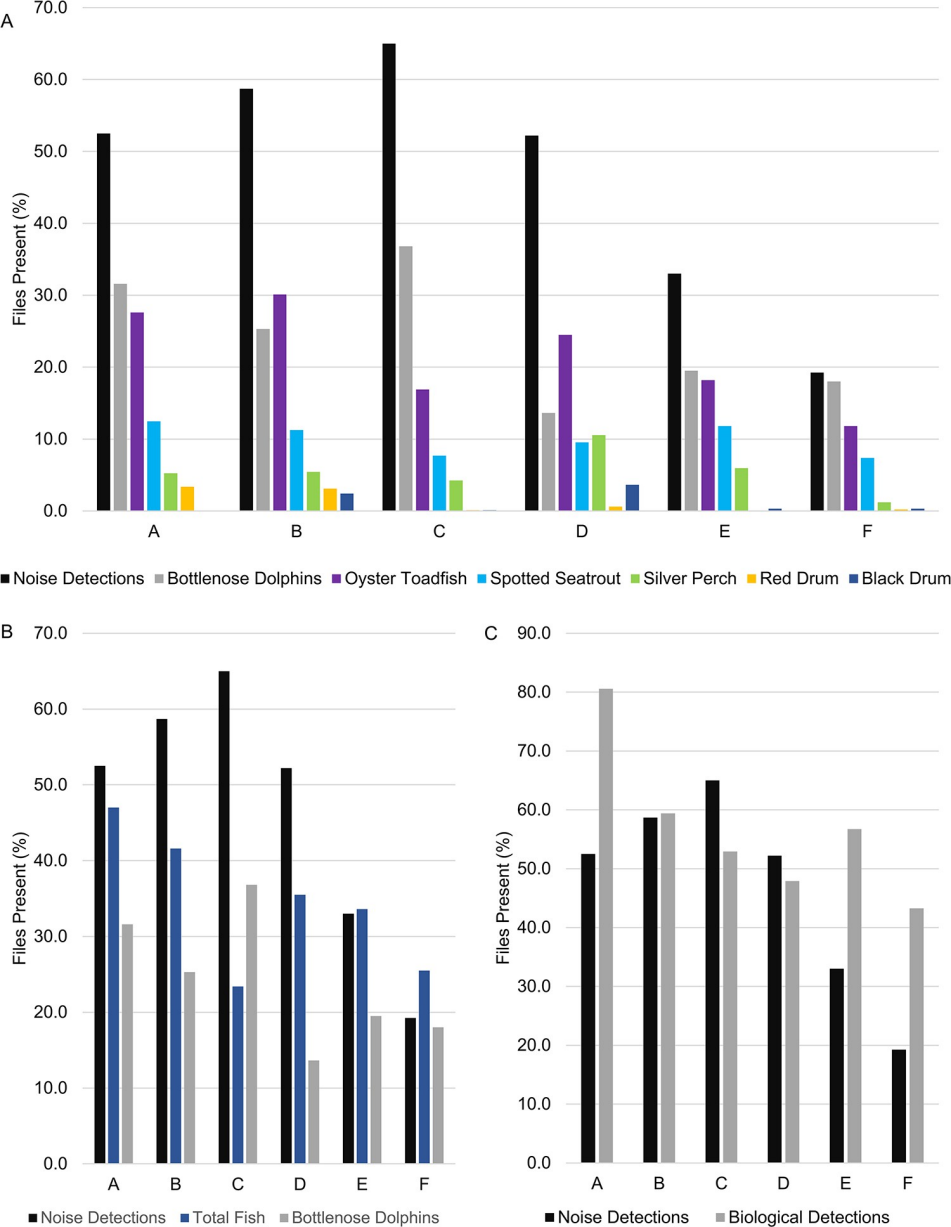
Prevalence of sound producers. Percent of files with (A) noise, bottlenose dolphin vocalizations, and species-specific fish calling; (B) noise, all fish calling, and bottlenose dolphin vocalizations; and (C) noise or biological sound (any fish call or dolphin vocalization). A = Wando River, B = Drum Island, C = SC Aquarium, D = Fort Sumter, E = Ashley River, and F = Citadel.

Snapping shrimp, seven fish species, two marine mammal species, an unknown biological sound, physical sounds associated with rain, and anthropogenic noise (*i*.*e*., recreational boats, ferries, and commercial vessels) were identified during manual review (Table 2). Snapping shrimp snaps were present in every acoustic file and their acoustic activity was represented by the high frequency SPL subset with anthropogenic noise detections removed (Fig 5). The identified fish species included oyster toadfish, spotted seatrout, silver perch, Atlantic croaker, red drum, weakfish (*Cynoscion regalis*), and black drum (Table 2). Oyster toadfish were the most frequently heard fish species, followed by spotted seatrout and then silver perch, with infrequent identification of black drum, red drum, Atlantic croaker, and weakfish (Table 2). Chorusing aggregations were detected for black drum, oyster toadfish, silver perch, spotted seatrout, and red drum; further statistical analysis was completed for these species (Table 3, Figs 6–9). Common bottlenose dolphins were detected across the estuary throughout the year (Fig 10). North Atlantic right whales were detected on 15 separate days during their migratory season in 2018 (detections occurred January 22 –March 9; Table 2, [85]). An unknown biological sound was also frequently detected at the Ashley River and Citadel stations, as well as the Wando River station (Table 2). This sound was presumably a fish call due to its frequency range (200–1200 Hz), similarity to other estuarine fish calls, and previous detections in the Chechessee Creek, SC [60]. Anthropogenic noise was the most frequently identified sound at every station across the estuary (Table 2, Fig 11), and physical sounds including rain occurred occasionally at every station (Table 2). Overall, the number of sound-producing organisms was consistent across the estuary, with the most frequent detections of biological sound occurring at the Wando River, and highest detections of anthropogenic noise at stations along the shipping channel (Wando River, Drum Island, SC Aquarium, and Fort Sumter) (Table 2, Fig 11).

**Table 2:** Prevalence of sounds at each station in Charleston Harbor from December 2017 to June 2019.

	A—Wando River		B—Drum Island		C—SC Aquarium		D—Fort Sumter		E—Ashley River		F—Citadel	
	Files with detections	Sum int.	Files with detections	Sum int.	Files with detections	Sum int.	Files with detections	Sum int.	Files with detections	Sum int.	Files with detections	Sum int.
**Snapping shrimp**	All files		All files		All files		All files		All files		All files	
**Fish**												
Oyster toadfish	3244 (27.6%)	6466	3513 (30.0%)	7633	1980 (16.9)	3732	2880 (24.5%)	5697	2137 (18.2%)	3773	1399 (11.8%)	2572
Spotted seatrout	1468 (12.5%)	3482	1315 (11.2%)	3216	899 (7.7%)	1955	1120 (9.5%)	2836	1389 (11.8%)	3208	876 (7.4%)	1791
Silver perch	616 (5.2%)	1386	635 (5.4%)	1354	497 (4.2%)	1264	1238 (10.5%)	3039	699 (5.9%)	1473	550 (4.7%)	1096
Atlantic croaker	663 (5.6%)	1350	73 (0.6%)	100	4 (0.0%)	6	3 (0.0%)	5	279 (2.4%)	486	746 (6.3%)	1408
Red drum	397 (3.4%)	781	364 (3.1%)	859	12 (0.1%)	24	71 (0.6%)	151	7 (0.1%)	14	18 (0.2%)	34
Weakfish	103 (0.9%)	199	23 (0.2%)	46	3 (0.0%)	3	18 (0.2%)	33	247 (2.1%)	331	119 (1.0%)	150
Black drum	10 (0.1%)	20	284 (2.4%)	564	11 (0.1%)	22	428 (3.6%)	903	36 (0.3%)	47	39 (0.3%)	58
Total fish detections	5522 (47.0%)		4870 (41.6%)		2736 (23.4%)		4168 (35.5%)		3951 (33.6%)		3010 (25.5%)	
**Bottlenose dolphins**												
Echolocation	3432 (29.2%)	28990	2700 (23.1%)	22652	4094 (35.0%)	37825	1405 (12.0%)	12059	2193 (18.6%)	15665	1716 (14.5%)	8175
Whistles	850 (7.2%)	6863	809 (6.9%)	8054	825 (7.1%)	5956	424 (3.6%)	3466	368 (3.1%)	3982	277 (2.3%)	1980
Burst pulses	554 (4.7%)	3750	145 (1.2%)	878	533 (4.6%)	2064	171 (1.5%)	1385	341 (2.9%)	1808	477 (4.0%)	1173
Sum of vocalizations	3712 (31.6%)	39603	2961 (25.3%)	31584	4306 (36.8%)	45845	1602 (13.6%)	16910	2289 (19.5%)	21455	2129 (18.0%)	11328
**Other**												
Unknown 1	4355 (37.0%)	8681	145 (1.2%)	229	200 (1.7%)	384	396 (3.4%)	791	2400 (20.4%)	4784	1727 (14.6%)	3424
Right whale	7 (0.06%)	7	11 (0.09%)	11	11 (0.09%)	11	11 (0.09%)	11	0 (0.0%)	0	0 (0.0%)	0
**Total Biological Detections**	9472 (82.8%)		6949 (59.4%)		6192 (52.9%)		5626 (47.9%)		6671 (56.7%)		5113 (43.3%)	
**Anthropogenic noise**	6169 (52.5%)		6872 (58.7%)		7602 (65.0%)		6129 (52.2%)		3886 (33.0%)		2276 (19.3%)	
**Rain**	160 (1.4%)		145 (1.2%)		160 (1.4%)		211 (1.8%)		197 (1.7%)		710 (6.0%)	
**Number of Files Reviewed**	11760		11699		11700		11752		11760		11822	

The number of 2-minute files with a call detected by each species is shown with the percentage (number of files divided by the total number of files analyzed at each station). The sum intensity (int.) was calculated by summing intensity scores for fish and summing the total number of counted dolphin vocalizations. Species include silver perch, oyster

toadfish, black drum, spotted seatrout, red drum, Atlantic croaker, weakfish, bottlenose dolphins, and North Atlantic right whales.

**Table 3:** Top ranked variable for each random forest model for black drum, oyster toadfish, silver perch, spotted seatrout, red drum, and bottlenose dolphin vocalizations. Blank cells indicate that variable was not included in the associated model. Fig 6 shows the relative rank of each factor.

	Station df = 5	Month / Temperature df = 11 / df = 1	Lunar phase df = 3	Tidal phase df = 3	Day / night df = 1	Noise presence* df = 1	Prediction accuracy / % variation explained**
**Black Drum**	Fort Sumter (D)	April	first quarter	high	night	0 = 1	98.93%
**Oyster Toadfish**	Drum Island (B)	April	full moon	high, rising, low	day	**0 > 1**	85.24%
**Silver Perch**	Fort Sumter (D)	April	first quarter, new moon	high	night	**0 > 1**	95.1%
**Spotted Seatrout**	Wando River (A), Drum Island (B), Ashley River (E)	May	full moon	high	night	0 = 1	91.81%
**Red Drum**	Wando River (A), Drum Island (B)	September	first quarter	rising	day	**0 > 1**	98.82%
**Bottlenose Dolphins**	SC Aquarium (C)	November-February	third quarter	falling	night	**1 > 0**	14.58%**

* 0 = no noise; 1 = noise present; bold and underlined values were confirmed in targeted models focused on species’ calling season and circadian pattern.

### 3.3 Snapping shrimp

Water temperature, station, lunar phase, day/night, and tidal phase all significantly influenced snapping shrimp acoustic activity (*i*.*e*., high frequency SPL with anthropogenic noise files removed) and explained 82.88% of the variance in the model (Table 1, Fig 2, p < 0.01). Snapping activity was highest in the Wando River and at Fort Sumter (Table 1, p < 0.001). Snapping activity had a positive relationship with temperature and peaked in July when temperatures were greatest (Table 1, Fig 5, p < 0.03). Acoustic activity of snapping shrimp occurred the least in January, when the water temperatures were the lowest (Fig 5). Snapping was highest during the first quarter and quietest during the third quarter of the lunar phase (Table 1, p < 0.001). More snapping activity occurred during nighttime than daytime (Table 1, p < 0.01). Tidal phase had the smallest impact on snapping and was greater during the high and low tide than the falling or rising tide (Table 1, p < 0.05). Though it was ranked low, the influence of tidal phase on snapping was evident in the heat map by the diagonal lines, which mirrored the tidal phases (Fig 4, S1 Fig).

### 3.4 Soniferous fish

Month/water temperature, station, day/night, anthropogenic noise, lunar phase, and tidal phase significantly influenced calling intensity of nearly all the sound-producing fish (Fig 6, p < 0.01). Fish models had a prediction accuracy between 85.2% - 98.9% (Table 3).

There were spatial and seasonal patterns of fish calling across Charleston Harbor. Fort Sumter and Drum Island were the major sites for black drum calling from late February to May (Tables 3 and 4, Fig 7). Calling peaked in April and occurred within a water temperature range of 14–24°C (Tables 3 and 4, Fig 7). Oyster toadfish were detected across the harbor, with the greatest calling intensity at Drum Island from March to June (Tables 3 and 4, Fig 7). Calling began when temperatures approached 15°C in the spring and stopped as temperatures reached 30°C in the summer (Tables 3 and 4, Fig 7). Silver perch were also detected across the harbor, with the greatest calling intensity detected at Fort Sumter between March and June (Tables 3 and 4, Fig 7). Calling peaked in April and occurred within a temperature range of 15–28°C (Tables 3 and 4, Fig 7). The Wando River, Drum Island, Ashley River, and Fort Sumter stations were hotspots for spotted seatrout calling, though consistent patterns of calling and chorusing were detected across the harbor from May through September (Tables 3 and 4, Fig 7). Calling occurred within a temperature range of 16–28°C with some calling starting in February and continuing to November (Tables 3 and 4, Fig 7). The Wando River, Drum Island, and Fort Sumter stations were the primary sites in which red drum calling and chorusing occurred from August to October (Tables 3 and 4, Figs 7 and 8). Calling began as temperatures started to decline from 30°C (Tables 3 and 4, Figs 7 and 8). The least amount of calling occurred from November to February for all fish species (S1 Table, Fig 7).

**Table 4:** Calling timelines and number of days chorusing (i.e. intensity score = 3) for fish species at each station.

Station	Year	Calling dates*	Temperature Range (°C)*	# days chorusing	Station	Year	Calling dates*	Temperature Range (°C)*	# days chorusing
**Black drum—*Pogonias cromis***					**Oyster toadfish—*Opsanus tau***				
A	2018	03/03–03/04	16.1–16.0	0	A	2018	02/22–10/29	16.7–20.2	8
	2019	no calling	no calling	0		2019	03/12 –na	16.1 –na	na
B	2018	02/24–04/28	17.4–20.4	0	B	2018	02/25–10/30	17.2–20.0	32
	2019	03/04–03/24	15.8–15.8	0		2019	03/04 –na	15.7 –na	na
C	2018	04/24–05/01	18.5–20.4	0	C	2018	02/28–11/29	15.4–20.6	2
	2019	no calling	no calling	0		2019	03/05 –na	15.5 –na	na
D	2018	02/25–05/07	18.3–22.3	12	D	2018	02/17–10/24	14.3–22.9	10
	2019	03/03–05/15	15.5–24.0	2		2019	02/28 –na	15.15 –na	na
E	2018	03/01–03/02	18.2–18.5	0	E	2018	01/01–12/24	9.8–13.1	0
	2019	no calling	no calling	0		2019	03/20 –na	16.1 –na	na
F	2018	03/16	14.1	0	F	2018	02/15–07/21	13.7–29.0	0
	2019	no calling	no calling	0		2019	03/16 –na	18.9 –na	na
**Silver perch—*Bairdiella chrysoura***					**Spotted seatrout—*Cynoscion nebulosus***				
A	2018	04/17–5/31	18.0–27.0	24	A	2018	04/17–11/30	18.8–13.9	126
	2019	04/06 –na	17.0 –na	na		2019	04/15 –na	20.7 –na	na
B	2018	04/18–05/20	17.8–25.0	9	B	2018	04/26–10/04	19.8–28.0	119
	2019	03/23 –na	15.5 –na	na		2019	03/15 –na	17.0 –na	na
C	2018	04/18–06/05	18.4–27.7	35	C	2018	04/18–09/27	18.4–28.7	52
	2019	04/06 –na	16.7 –na	na		2019	04/13 –na	20.9 –na	na
D	2018	03/30–06/09	16.4–27.9	45	D	2018	04/03–11/20	16.6–17.2	135
	2019	03/11 –na	16.8 –na	na		2019	03/14 –na	17.0 –na	na
E	2018	03/19–05/31	15.3–27.1	18	E	2018	04/23–10/08	18.6–28.0	127
	2019	03/13 –na	16.3 –na	na		2019	03/01 –na	15.0 –na	na
F	2018	03/20–05/27	16.7–26.7	0	F	2018	04/26–10/29	20.9–19.0	22
	2019	03/10 –na	17.9–27.4	na		2019	04/12 –na	21.1 –na	na
**Red drum—*Sciaenops ocellatus***									** **
A	2018	07/31–12/20	29.3–13.4	0					
B	2018	07/17–10/19	29.7–25.7	58					
C	2018	09/06–09/24	29.9–28.6	0					
D	2018	07/24–10/23	29.6–22.9	7					
E	2018	09/30–10/15	28.8–26.7	0					
F	2018	06/21–11/17	30.7–16.0	0					

*Includes an intensity score of 2 or 3

na = data are not available based on the end recording date of 06/02/2019

Lunar, day/night, and tidal cycles also significantly influenced fish calling (Table 3, Fig 6). Spotted seatrout and oyster toadfish called more frequently during the full moon (Table 3). Silver perch, black drum, and red drum, however, called more frequently during the first quarter of the lunar cycle (Table 3). Spotted seatrout, silver perch, and black drum called and chorused more at night, while oyster toadfish and red drum called and chorused more during the day (Table 3, Fig 8). Like snapping activity, fish calling tended to be greatest during the high tide (Table 3, S1 Fig, p < 0.05).

The presence of anthropogenic noise negatively affected the calling of some fish species (Table 3, Fig 9, p < 0.001). When targeted fish models focused on the respective calling season and circadian pattern, anthropogenic noise negatively affected calling of silver perch, oyster toadfish, and red drum (Table 3, p < 0.01).

### 3.5 Bottlenose dolphins

Bottlenose dolphin vocalizations were influenced by station, water temperature, lunar phase, day/night, tidal cycle, and the presence of anthropogenic noise (Table 3, Fig 6, p < 0.01). This model explained a relatively low amount of variance compared to the other models, with only 14.6% of the variance explained (Table 3). The SC Aquarium had the highest number of vocalizations (Table 3, Figs 10 and 11, p < 0.001). Vocalization patterns displayed an inverse relationship with temperature, with more vocalizations during the colder months (Table 3, Fig 10). Generally, vocalizations peaked in winter months when temperatures were lowest and then decreased as the estuary warmed; vocalizations were lowest from late spring through the summer (S1 Table, Fig 10, p < 0.01). Lunar patterns showed that vocalizations were greatest in the third quarter of the lunar cycle (Table 3, p < 0.001). More bottlenose dolphin vocalizations were detected at night (Table 3, p < 0.001). Tidal cycles showed significant patterns, with higher vocalizations on the falling tide compared to the rising tide (Table 3, p < 0.001). Bottlenose dolphin vocalizations increased with anthropogenic noise, an opposite pattern that was observed in fish (Table 3, p < 0.001). This relationship was confirmed in targeted models that removed seasonality to limit collinearity (Table 3, p < 0.01).

### 3.6 Anthropogenic noise detection

Anthropogenic noise detections were significantly influenced by station, month, weekday, and day/night cycles, with these variables resulting in a 72.5% prediction accuracy for the model (Table 1, Fig 2, p < 0.01). The highest number of anthropogenic noise detections occurred at the SC Aquarium with the least detections at the Ashley River and Citadel stations (Table 1, Fig 11, p< 0.001). These detections illustrate an obvious spatial pattern, with more vessel noise along the shipping channel (Fig 11). Though anthropogenic noise from a single ship could have been detected at multiple stations along the shipping channel, each station had a significantly different amount of noise detections (Table 1, p < 0.05). Anthropogenic noise prevalence followed strong temporal patterns associated with temperature, with the highest detections occurring with increased temperatures in the summer months and less detections when temperatures decreased in the winter months (Table 1). The day of the week significantly influenced anthropogenic noise detections (Table 1). Weekends had higher anthropogenic noise occurrences than weekdays, with the most noise detected on Saturdays followed by Fridays and Sundays (Table 1, p < 0.001). The day/night circadian cycle was the most important variable for anthropogenic noise occurrence and was more prevalent during the day than at night (Table 1, p < 0.001).

### 3.7 Quantitative contributions of biological sound and anthropogenic noise to the soundscape

**Fig 12:**
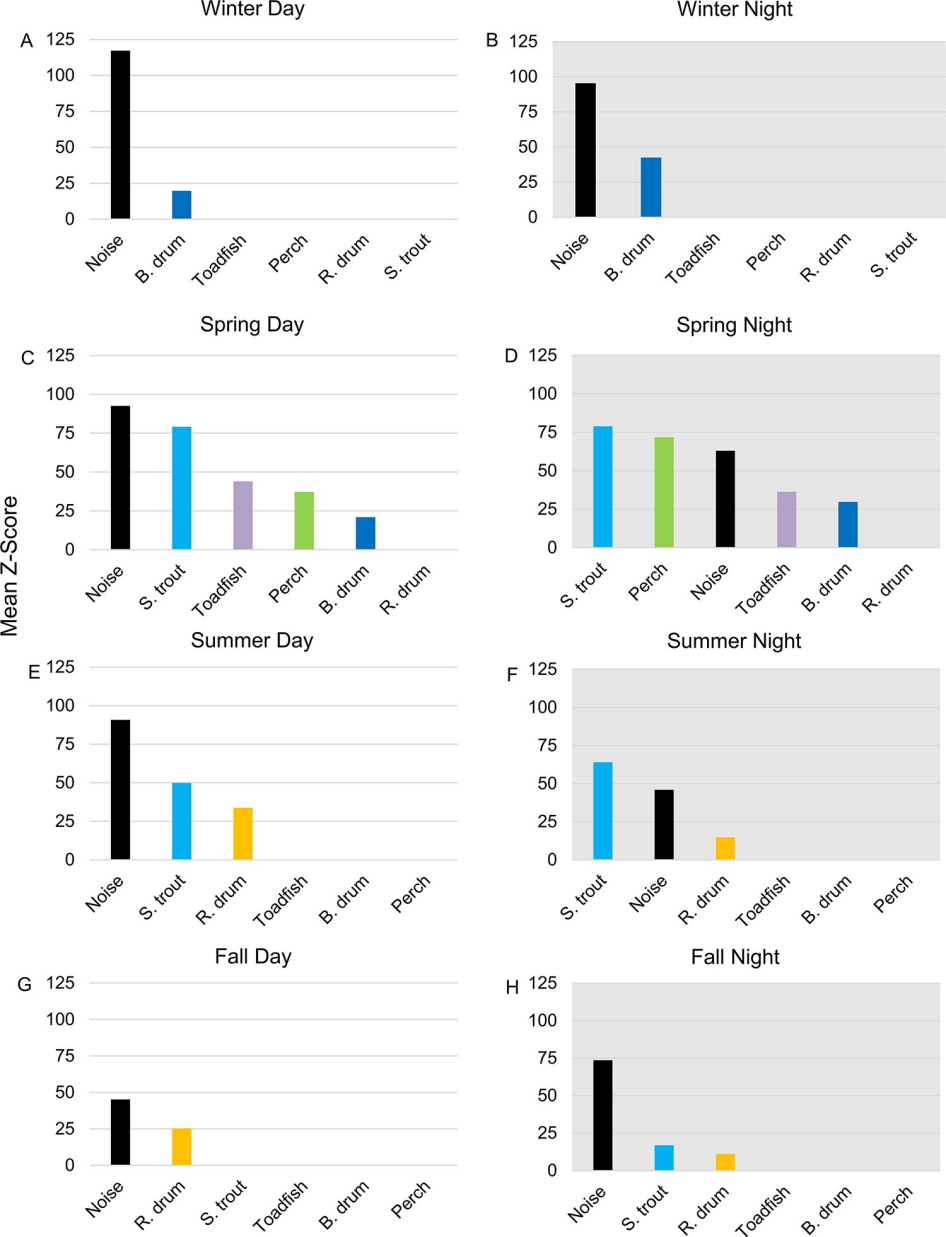
Sound producer contribution to sound pressure levels. Sound producer contributions from anthropogenic noise, black drum, oyster toadfish, silver perch, red drum, and spotted seatrout to low (50–1200 Hz) frequency, received sound pressure levels per season and day/night cycle. (A) Winter day, (B) winter night, (C) spring day, (D) spring night, (E) summer day, (F) summer night, (G) fall day, and (H) fall night. Bars represent the significance (mean Z-score) of each sound producer from the Boruta wrapper algorithm based on a random forest model. All the factors were significant at p < 0.01. Day and night were classified by daily sunrise and sunset times. Seasons were applied with the astronomical start dates: spring begins March 20, summer begins June 21, fall begins September 22, and winter begins December 21.

During winter days and nights, anthropogenic noise was the greatest contributor to low frequency SPLs with additional contribution from black drum (Fig 12). During spring days, anthropogenic noise contributed the most to low frequency SPLs followed closely by spotted seatrout; other spring-time sound producers contributed less substantially to low frequency SPLs (Fig 12). At night, spotted seatrout and silver perch calling outranked anthropogenic noise (Fig 12). Summer days were dominated by anthropogenic noise, but spotted seatrout calling outranked anthropogenic noise during summer nights (Fig 12). Patterns of spotted seatrout chorusing contributed the most to low frequency SPLs compared to all fish species as highlighted by the oscillating nightly, chorusing pattern displayed in the low frequency SPL heat map (Figs 3 and 8). Anthropogenic noise ranked as the top sound contributor during the fall, with red drum contributions during the day and both spotted seatrout and red drum contributions at night (Fig 12).

High frequency SPLs without anthropogenic noise were well described by water temperature, illustrating that snapping shrimp were the main acoustic contributor to this range. In the sound contributor random forest models, anthropogenic noise was also found to significantly influence high frequency SPLs for all seasons and day/night cycles, with a small contribution from bottlenose dolphins during winter nights as well as fall days and nights when their vocalizations increased (p < 0.01, data not shown).

## 4. Discussion

### 4.1 Spatial and temporal patterns of biological sound

#### 4.1.1 Snapping shrimp

High snapping shrimp activity in Charleston Harbor was detected at the Wando River and Fort Sumter stations, which contain oyster reef or rocky substrate habitats. Oyster reefs provide habitat structure and resources for benthic species like snapping shrimp [23, 30]. The Wando River station was located near oyster reefs bordering the entrance of Hobcaw Creek, while Fort Sumter contains a perimeter of large, submerged rocks that provide habitat for snapping shrimp. This finding is similar to the May River and Chechessee Creek, SC where snap rates were highest near oyster reefs and anti-erosion structures, respectively [23, 60].

Seasonal patterns of elevated snapping (indicated by increased high frequency SPLs excluding noise) in the summer months throughout Charleston Harbor were consistent with other snapping shrimp and soundscape studies conducted in Charleston Harbor, the May River, and Chechessee Creek, SC, and in the West Bay Marine Reserve estuary (WBMR) and Middle Marsh, North Carolina (NC) [23, 60, 61, 79, 86]. Snapping shrimp in Charleston Habor have been found in male-female pairs year-round and are reproductively active from at least April to November, matching the seasonal patterns established in this study [87]. Furthermore, snap rate in the WBMR was strongly correlated with water temperature; this variable explained 65–86% of the variance in snap rates [86]. These results are comparable with the temperature model in the current study that explained 82% of the variance in snapping activity and explains why the percent of explained variance is considerably higher for high frequency SPLs than low frequency SPLs.

In Charleston Harbor, circadian rhythms of snapping behavior match those established in other estuaries in North and South Carolina [4, 23, 60, 79, 86]. In general, snapping shrimp tend to be more active at night [88]. In the summer months, snapping activity was higher at night in Charleston Harbor; it was also higher during summer nights in the May River, Chechessee Creek, Middle Marsh, and the WBMR [23, 60, 79, 86]. In addition, the May River and WBMR exhibited a diurnal shift in high frequency SPLs that reflected increased snapping shrimp activity during the day in winter, spring, and fall [23, 86]. This pattern was also observed in the high frequency SPL heat map in Charleston Harbor (Fig 4). These shifts in behavior could be related to differences in light availability, prey dynamics, or metabolic responses from summer to winter [86].

Charleston Harbor exhibited different lunar and tidal patterns of snapping activity than patterns observed in the May River, Chechessee Creek, and Middle Marsh. In Charleston Harbor, lunar differences in the high frequency SPL range were observed only when anthropogenic noise files were removed from the dataset. Snapping activity was higher during the first quarter of the lunar cycle, whereas in the May River and Chechessee Creek, high frequency SPLs and snapping activity was increased on the new moon [23, 60]. In Middle Marsh, high frequency SPLs did not have any significant differences between lunar cycles [79]. In Charleston Harbor, snapping activity was higher on the high tide as compared to snapping observed during other tidal phases. These tidal patterns are opposite to those identified in the May River and Chechessee Creek, which exhibited higher snapping activity during low tide [23, 60]. The differences in patterns between Charleston Harbor versus the May River and Chechessee Creek may be associated with dissimilar habitats (*i*.*e*., degraded and smaller oyster reefs in Charleston Harbor, smaller tidal range in Charleston Harbor) and / or masking by anthropogenic noise [89]. Patterns in Middle Marsh found no tidal differences in high frequency SPLs [79]. These differences may occur because oyster reefs in the southern portion of SC are intertidal (*i*.*e*., dry on low tide), while oyster reefs in NC are subtidal (*i*.*e*., submerged on low tide).

#### 4.1.2 Soniferous fish

Spatial distribution patterns of fish calling were associated with distance to deep water, habitat structure, and salinity. Soniferous fish chorus to attract mates to spawning aggregations, and these aggregations occur more frequently in deeper water [3, 37, 74, 90]. In Charleston Harbor, the stations along the shipping channel, particularly the Wando River, Drum Island, and Fort Sumter stations, were closer to deeper water and were more likely to detect calling and choruses of spawning aggregations. Black drum and oyster toadfish tended to have higher calling intensity scores in areas of higher habitat structure; silver perch called more frequently where salinity was highest; red drum tended to call more in areas of greater depth; and spotted seatrout were detected throughout the estuary.

Black drum calling occurred infrequently across the estuary. Black drum inhabit offshore ecosystems for most of the year and return inshore during late winter to spawn [91]. Because black drum are bottom feeders, these fish prefer habitats with structure where prey can be found [92]. In Charleston Harbor, the longest black drum calling timelines occurred at Drum Island and Fort Sumter; chorusing only occurred at Fort Sumter. Drum Island is near the Ravenel Bridge, and Fort Sumter is an island with rocky structure, both of which provide suitable habitat for this species. Seasonally, peak spawning for black drum occurs during February and March throughout the Gulf of Mexico, which matched the peak calling season detected in this study [91–93]. In other studies, black drum spawning peaked from 18–22°C, with no sound production at temperatures less than 15°C [36, 90, 94, 95]. These temperature ranges matched those found in the current study; the lowest water temperature recorded with calling was 15.5°C, and the chorusing patterns identified at the Fort Sumter station occurred within a temperature range of 18.3–22.3°C.

Oyster toadfish calling exhibited high levels of spatial synchrony and were identified across all stations. Oyster toadfish utilize offshore muddy habitats to overwinter and then move into oyster reefs and structured habitats during the spring to create nests for spawning [96, 97]. Chorusing occurred primarily at Drum Island followed by Fort Sumter, illustrating the preference for highly structured habitats suitable for nesting and feeding [79, 96, 98]. In the present study, seasonal patterns of oyster toadfish calling increased in the spring, matching established spawning patterns in other geographical locations [96, 97, 99, 100]. Oyster toadfish differed from other soniferous fish by calling throughout the day, which was consistent with findings from other acoustic studies [99, 101].

Silver perch calling was identified across the estuary with chorusing identified at every station except for the Citadel. This species is ubiquitous throughout southeastern U.S. salt marsh estuaries [102]. Silver perch have been shown to spawn in higher salinity locations, indicating why chorusing was greatest at the Fort Sumter station, where salinity was highest across the estuary [37]. Studies show that silver perch spawning occurs between March and June at temperatures less than 26°C in this region [36, 37, 103, 104]. In this study, chorusing matched this timeline and temperature range with calling beginning in March or April and ending in June, when temperatures exceeded 26°C.

Spotted seatrout displayed the most consistent, spatial patterns across the estuary, with calling and chorusing at every station. A study using passive acoustics to identify spawning locations in Charleston Harbor in 1992 found that the most intense chorusing occurred at Drum Island [21]. In the present study, spotted seatrout calling was also high at Drum Island. In South Carolina, spotted seatrout spawn from April through September at temperatures above 23°C [105, 106], and these fish have been shown to call in a variety of dissolved oxygen, salinity, and depth ranges [21, 37, 105, 107, 108]. In fact, spotted seatrout have displayed the ability to adapt egg size to remain neutrally buoyant in a variety of salinities [109]. Spotted seatrout calling in this study matched expected temporal and temperature patterns and their large tolerance in conditions may explain the prevalence of chorusing across Charleston Harbor.

Low levels of red drum calling were identified at every station, but chorusing aggregations primarily occurred at Drum Island (58 days of chorusing detected) with a small amount detected at Fort Sumter (7 days of chorusing detected). Fort Sumter is closest to the deepest section of the Charleston Habor [110], where red drum spawning aggregations have previously been found [22]. Red drum have been shown to prefer deep water for spawning aggregations (as detected by chorusing) but call over a wide range of salinities [37, 111]. It is possible that red drum utilize the depth of the shipping channel throughout Charleston Harbor for spawning activities despite the decreased salinity further from the mouth of the harbor. Seasonally, red drum spawning occurs from August to October, with a temperature range of 30–25°C [22, 37, 90, 112]. The results of this study matched these findings, though the temperature range of chorusing was narrower at 30–27°C.

Seasonally, low frequency SPLs (without anthropogenic noise) reflected patterns of fish calling. The increase in fish sound production as temperatures warmed in the spring, and the decrease in sound production as temperatures fell in the fall, matched the overall patterns found in the May River, Chechessee Creek, and Middle Marsh soundscapes [4, 23, 60, 74, 79]. In general, Sciaenids began calling shortly before sunset and chorused into the evening hours, elevating low frequency SPLs during spring and summer nights. These circadian rhythms match those established in other estuaries in the southeastern U.S. [2, 74, 79, 112–114]. Similar to the May River and Chechessee Creek soundscapes, spotted seatrout nightly chorusing followed an oscillating pattern observed in the low frequency SPL heat maps, and this pattern was associated with the lunar cycle [23, 60]. However, in Charleston Harbor, the extended nightly chorusing of spotted seatrout occurred on the full moon, while in the May River and Chechessee Creek, these extensions occurred on the first and third quarters of the lunar cycle [4, 23, 60, 61]. Similar to Charleston Harbor, the Middle Marsh soundscape exhibited higher low frequency SPLs and fish calling during the full moon [79].

Low frequency SPLs and fish calling were greatest on the high tide, which matched the tidal patterns discovered in Middle Marsh and the May River [79, 115]. Evidence exists that silver perch, spotted seatrout, and red drum exhibit behaviors to enhance retention of early life stages in nursery habitats. Fertilized eggs float, and eggs of these species quickly hatch 18–30 hours after fertilization [21, 107, 116–118]. It is obvious that eggs are passive and drift from the original spawning location due to currents and tidal movements [107, 117, 118]. The discovery that fish chorus more frequently on the rising and high tide provides some evidence that this type of spawning behavior may retain eggs within the estuary (S1 Fig).

#### 4.1.3 Bottlenose dolphins

Bottlenose dolphin vocalizations were greatest at the mouth of the Wando and Cooper Rivers with elevated vocalizations at the SC Aquarium, Wando River, and Drum Island stations, especially during the winter, illustrating that these locations may be important foraging areas. Interestingly, the Wando River and Drum Island also had the highest intensity of fish calling across the estuary. Sciaenids comprise a large percentage of the bottlenose dolphin diet in SC [41, 42]. The high levels of Sciaenid calling at these locations may indicate increased fish abundance at these locations. In the May River, dolphin vocalizations had the highest detections at the mouth of the river, where fish calling was also greatest [23, 76]. Greater dolphin vocalizations have also been recorded at the mouth of Galveston, Texas and Tampa Bay, Florida [119–121]. In addition, estuaries across the Gulf of California have found increased foraging of bottlenose dolphins near the mouth [122]. Recent studies suggest prey become concentrated at the mouth of estuaries, and these locations function as predation hotspots [123].

Seasonal, circadian, and tidal patterns of bottlenose dolphin vocalizations were similar to those found in the May River [23, 76]. Vocalizations peaked in winter months when fish calling was lowest, matching those patterns found at the mouth of the May River [23, 76]. One hypothesis for this general pattern is that bottlenose dolphins may use passive listening to find prey during the Sciaenid calling season but need to increase their use of echolocation during times when fish calling is not occurring [9, 76, 124]. Another hypothesis is that during the winter, prey is scarce, and dolphins may need to echolocate more frequently to find prey.

Similar to the May River soundscape, dolphin vocalizations increased at night [76]. In Charleston Harbor, tidal patterns revealed acoustic activity of bottlenose dolphins was greater on the falling tide as compared to activity on the rising tide, a pattern that was also observed at the mouth of the May River [76]. Bottlenose dolphins may increase their foraging efforts as fish such as spotted seatrout and red drum move from the shelter of *Spartina* on high tide to deeper channels on the falling tide [76, 125]. Higher vocalizations on the falling tide could also simply indicate movement of bottlenose dolphins closer to acoustic recorders due to decreasing access to smaller channels with the ebbing tide.

### 4.2 Spatial and temporal patterns of anthropogenic noise

Anthropogenic noise detection occurred most frequently at stations along the shipping channel. This finding indicates there is either less vessel activity in the Ashley River as compared to activity in the Cooper and Wando Rivers or that the noise from container ships are heard at multiple stations throughout the shipping channel as they travel through Charleston Harbor. Temporally, anthropogenic noise was detected most often during the summer, on the weekends, and during the daytime. This finding matches the temporal patterns of anthropogenic noise detection in the May River [23, 53]. Shipping terminals in Charleston Harbor operate from Monday-Saturday year-round, with ships arriving throughout the week and at all hours of the day [126, 127]. Therefore, in Charleston Harbor, the heightened anthropogenic noise detection during these popular recreational times illustrates that recreational boat traffic is still a significant source of anthropogenic noise across the harbor. Additionally, consistent daily anthropogenic noise detection occurred from 10:00–16:00 at the SC Aquarium and Fort Sumter stations. This pattern is displayed in the low and high frequency heat maps and coincides with the ferry schedule that travels between these two stations from 10:00–16:00 year-round and extends until 18:00 from March to August [128].

### 4.3 Influences of human activities on the soundscape

#### 4.3.1 Behavioral response of biological sound producers to anthropogenic noise

Fish calling intensity decreased with anthropogenic noise detections for silver perch, oyster toadfish, and red drum, illustrating the potential for auditory masking of communication signals for these species. Vessel noise overlapped with the frequency ranges of all fish species investigated in this study, as was found in the May River [53]. Anthropogenic noise from commercial ships elicits a peak frequency 20–200 Hz and can radiate to frequencies of up to 100 kHz [129–131]. Small vessels with outboard or inboard engines have a peak frequency from 100 Hz—7 kHz and can radiate up to 40 kHz [53, 132, 133]. Since the shipping channel in Charleston Harbor is perhaps a more favorable spawning habitat due to its depth, and vessel noise peaks in the same range frequency range as fish calling, it is likely shipping activities during early evening hours masks fish acoustic signals. This masking could decrease the likelihood of females finding spawning aggregations, which could in turn reduce reproductive success [2, 37–39, 53]. Soniferous fish that call and communicate during the day, like oyster toadfish and red drum, are more susceptible to recreational and commercial vessel noise disturbance, since operation of these boats and ships primarily occur during the day. Red drum have been previously shown to be at high risk for auditory masking of signals due to their earlier chorusing times in the May River [53]. Lusitanian toadfish (*Halobatrachus didactylus*) signals in the Tagus River estuary, Portugal, have also been shown to experience masking by ship noise [134].

Bottlenose dolphins increased the rate of their vocalizations when vessels were detected. The positive correlation between the number of vocalizations and anthropogenic noise suggests that bottlenose dolphins may increase their vocalization rates to overcome masking of their signals [135]. Bottlenose dolphins have been shown to acclimate to noisy environments by altering their whistle form, duration, and frequency in other regions [49, 136, 137], but this additional vocal effort can also increase their metabolic rate [55, 138–140]. This cost has cumulative energetic consequences when combined with energy expenditure involved in failed foraging events, physiological stress response, and anthropogenic noise avoidance behaviors [55].

Interestingly, the increased identification of bottlenose dolphin vocalizations along the shipping channel could indicate that dolphins are not avoiding areas with high vessel activity. Detections of fish calling were greater along the shipping channel, so it is likely that dolphins followed their prey despite increased anthropogenic activity. Studies in the Shannon Estuary, Ireland, and the Swan-Canning Rivers estuary, Australia, also showed that bottlenose dolphins (*Tursiops truncatus* and *Tursiops aduncus*, respectively) used heavily trafficked recreation and shipping areas [141, 142]. In fact, bottlenose dolphins in Charleston Harbor have displayed “shipside feeding” where these animals use the side of docked commercial ships to trap fish [143]. Because most bottlenose dolphin vocalizations occur at higher frequencies (typically recorded above 7 kHz in this study), it is possible their acoustic signals are disrupted more by recreational boats that produce high frequency noise as compared to commercial ships that primarily produce lower frequency noise at slower speeds [129, 133, 144].

#### 4.3.2 Masking of biological soundscape patterns by anthropogenic noise

Despite high anthropogenic noise influence, low frequency SPLs coarsely reflected the seasonal, lunar, and tidal patterns of fish calling. During spring and summer nights, fish calling (*i*.*e*., black drum, silver perch, oyster toadfish, spotted seatrout, and red drum) dominated the soundscape, and these patterns were detected both in manual review and low frequency SPL values. However, the low frequency biological SPL patterns in Charleston Harbor were less obvious due to increased vessel detections, which was quite different than the distinct patterns observed in the May River and Chechessee Creek soundscape reported in other studies [23, 60]. Additionally, in Charleston Harbor, circadian cycles of increased nighttime chorusing were overshadowed by increased anthropogenic noise prevalence during the daytime in the full SPL dataset, and biological circadian patterns were only identified when anthropogenic noise files were excluded. These findings indicate that SPL patterns are limited in their interpretation of biological activity for noisy regions and that the overall acoustic signature that we find in more pristine estuaries is lost in Charleston Harbor [23, 60, 61, 79].

High frequency SPL patterns were less pronounced in the urbanized estuary of Charleston Harbor than less impacted estuaries of the May River and Chechessee Creek [23, 61]. The high frequency SPL dataset reflected seasonal and lunar patterns of snapping activity but only revealed circadian and tidal patterns when anthropogenic noise files were excluded. Again, the loss of these tidal and circadian patterns indicates heavier influence of anthropogenic noise in Charleston Harbor than surrounding estuaries.

Sound pressure levels are important indicators of biological patterns in soundscape studies [77–79]. Low frequency SPLs have been used to represent patterns of fish calling, and high frequency SPLs have been used to represent snapping shrimp acoustic activity [26, 78, 79]. However, in this study, fine-scale biological patterns were not clearly identified in SPL patterns until files with anthropogenic noise presence were removed. Notably, both low and high SPLs with anthropogenic noise included in the dataset were louder during the day reflecting the increased presence of anthropogenic noise detection during the day. When anthropogenic noise files were removed, low and high SPLs were louder at night, which reflects the increased acoustic activity of fish and snapping shrimp that is normally observed during this time in less impacted estuaries [23, 60, 61]. This limits the interpretation of SPLs in more urbanized, noisy estuaries and further indicates that the overall acoustic signature that we find in more pristine estuaries is lost in Charleston Harbor.

### 4.4 Conclusions

This study is the first to characterize the Charleston Harbor soundscape, providing an illustration of how chronic, anthropogenic noise in an urbanized port masks biological sounds and alters soundscape patterns normally detected in more pristine estuaries. Anthropogenic noise was prevalent across Charleston Harbor, especially along the shipping channel (*i*.*e*., Wando River, Drum Island, SC Aquarium, and Fort Sumter stations) and influenced the estuarine soundscape. Even though this estuary had high shipping traffic and anthropogenic noise across the 1.5 years of data collection for this study, the heightened anthropogenic noise detection on the weekends and during the summer illustrate that recreational boat traffic still comprises a significant percentage of anthropogenic noise across the harbor. In Charleston Harbor, circadian cycles of increased nighttime fish chorusing were overshadowed by increased anthropogenic noise prevalence during the daytime, and biological circadian patterns were only identified when anthropogenic noise files were excluded. These findings indicate that SPL patterns are limited in their interpretation of biological activity for noisy regions and that the overall acoustic signature that we find in more pristine estuaries is lost in Charleston Harbor. Interestingly, behavioral responses to anthropogenic activity varied among trophic levels. Calling intensity of silver perch, oyster toadfish, and red drum decreased when vessels were present. Alternatively, bottlenose dolphin vocalizations increased in the presence of anthropogenic noise.

Biological sound followed similar spatial patterns across Charleston Harbor with high levels of snapping shrimp acoustic activity, fish calling/chorusing, and bottlenose dolphin vocalizations detected at the Wando River and Drum Island stations (along the shipping channel), and low levels of each sound producer at the Citadel station. Because the shipping channel is favorable for fish spawning aggregations due to its depth and commercial vessels produce noise in the same frequency range, there is potential for impacts to reproductive output of soniferous fish species. However, because anthropogenic noise is more commonly detected during the day, the threat of masking may be minimal for species with consistent nighttime chorusing like black drum and spotted seatrout but more severe for oyster toadfish and red drum that chorus during the day. Future studies could explore differences in the abundance of sound-producing fish between urbanized and less impacted estuaries to elucidate the impacts of anthropogenic noise on fish reproduction.

## Supporting information

S1 Fig Heat map of fish chorusing with tidal phase background. Spatial and temporal patterns of fish chorusing in Charleston Harbor. Time is shown between noon and noon the next day at (A) Wando River, (B) Drum Island, (C) SC Aquarium, (D) Fort Sumter, (E) Ashley River, and (F) Citadel stations. Background color represents the tidal phase. White spaces represent gaps in the data due to maintenance of equipment between deployments.
(TIF)

S1 Table Significant differences in post-hoc testing for root mean square (rms) sound pressure levels (SPLs) (with and without noise included), noise detections, fish calling, and bottlenose dolphin vocalizations. Stations include (A) Wando River, (B) Drum Island, (C) SC Aquarium, (D) Fort Sumter, (E) Ashley River, and (F) Citadel. Greater than (>) and less than (<) symbols indicate the rank of each variable in post-hoc testing. Blank cells indicate that variable was not included in the associated model. * 0 = no noise; 1 = noise present; bold and underlined values were confirmed in targeted models focused on species’ calling season and circadian pattern.
(DOCX)

S1 Data (CSV)
