# Supplementary figures and images for: The Biological and Anthropogenic Soundscape of an Urbanized Port – the Charleston Harbor Estuary, South Carolina, USA
Source: PLoS One. 2023 Apr 19;18(4):e0283848. doi: 10.1371/journal.pone.0283848 (PMC10115300; doi:10.1371/journal.pone.0283848)

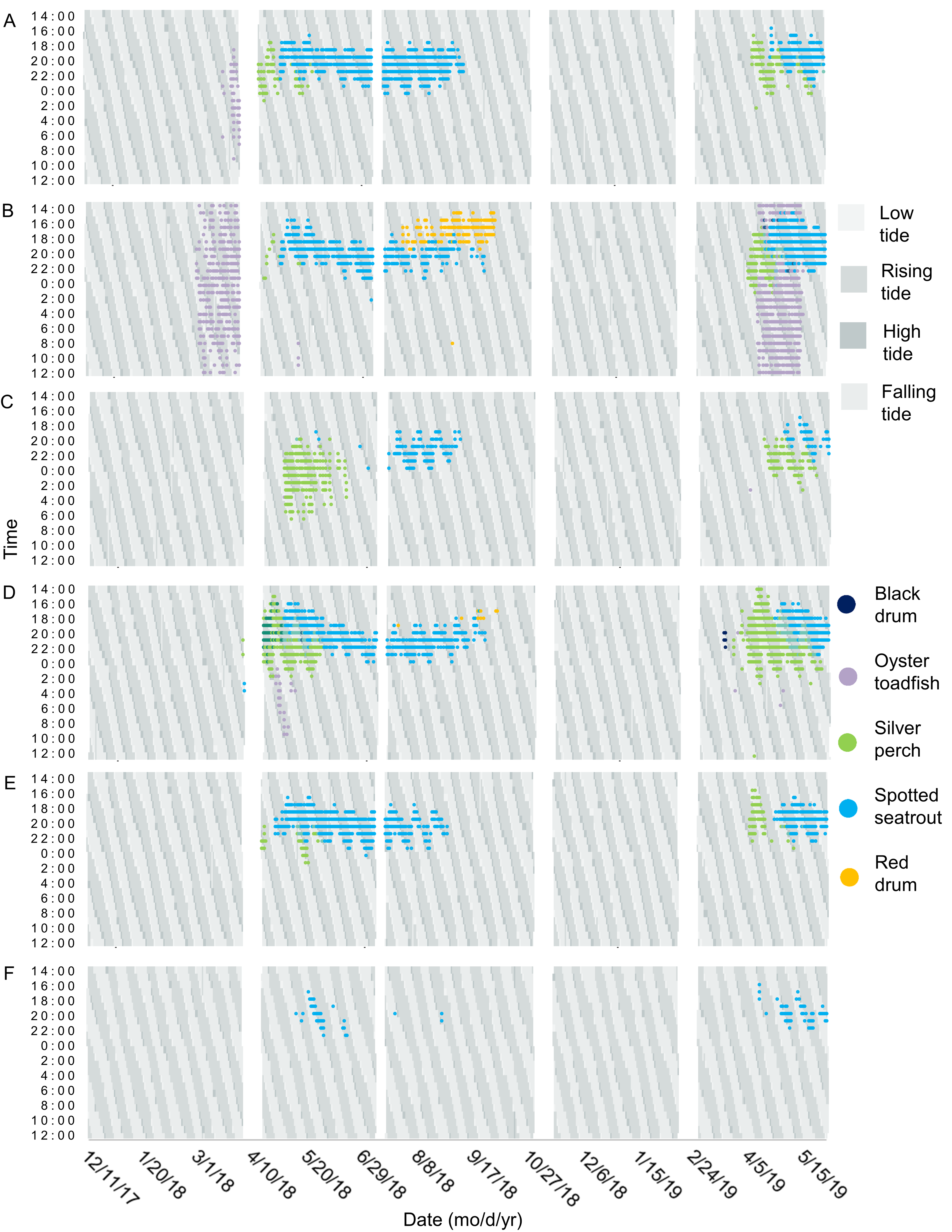

Supplement: S1 Fig — Spatial and temporal patterns of fish chorusing in Charleston Harbor. Time is shown between noon and noon the next day at (A) Wando River, (B) Drum Island, (C) SC Aquarium, (D) Fort Sumter, (E) Ashley River, and (F) Citadel stations. Background color represents the tidal phase. White spaces represent gaps in the data due to maintenance of equipment between deployments. (TIF) [file pone.0283848.s001.tif]
