# Supplementary material for: The Biological and Anthropogenic Soundscape of an Urbanized Port – the Charleston Harbor Estuary, South Carolina, USA
Source: PLoS One. 2023 Apr 19;18(4):e0283848. doi: 10.1371/journal.pone.0283848 (PMC10115300; doi:10.1371/journal.pone.0283848)
Supplement: S1 Table — Stations include (A) Wando River, (B) Drum Island, (C) SC Aquarium, (D) Fort Sumter, (E) Ashley River, and (F) Citadel. Greater than (>) and less than (<) symbols indicate the rank of each variable in post-hoc testing. Blank cells indicate that variable was not included in the associated model. * 0 = no noise; 1 = noise present; bold and underlined values were confirmed in targeted models focused on species’ calling season and circadian pattern. (DOCX) [file pone.0283848.s002.docx]

**S1 Table.** **Significant differences in post-hoc testing for root mean square (rms) sound pressure levels (SPLs) (with and without noise included), noise detections, fish calling, and bottlenose dolphin vocalizations. Stations include (A) Wando River, (B) Drum Island, (C) SC Aquarium, (D) Fort Sumter, (E) Ashley River, and (F) Citadel. Greater than (>) and less than (<) symbols indicate the rank of each variable in post-hoc testing. Blank cells indicate that variable was not included in the associated model.**

| **Random Forest Model** | **Station** | | **Month** | | **Lunar phase** | **Tidal phase** | **Day/ night** | **Noise presence*** | | | **Weekday** |
| --- | --- | --- | --- | --- | --- | --- | --- | --- | --- | --- | --- |
| **Low Freq. rms SPL 50-1200 Hz** | | B>D>E>C>A>F | | May, Jun > Jul > Apr, Aug > Sep, Oct > Mar > Nov, Dec, Jan, Feb | full > first, third > new | high > rising > falling > low | day > night |  | | |  |
| **High Freq. rms SPL 7-40 kHz** | C>A>D>E>B>F | | | Jul > Jun, Aug, Sep > Oct > May > Apr > Nov > Mar > Dec > Feb > Jan | first > full > new, third | no differences | day > night |  | | |  |
| **Noise Detection** | C>B>D,A>E>F | | | Jun > Feb, March, April, May, July, Aug > Jan, Sep, Oct, Nov, Dec |  |  | day > night |  |  | Sat > Sun, Fri > Wed, Thurs > Mon, Tues | |
| **Low SPL - No Noise** | B>D,E>A>C>F | | | May, Jun, Jul > Apr > Aug > Sep > Oct > Mar > Nov, Dec, Jan, Feb | full > first, third, new | high > rising, low > falling | night > day |  | | |  |
| **High SPL - No Noise** | A>D>C>E>B>F | | | Jul > Jun, Aug, Sep, Oct > May > Apr > Nov > Mar > Dec > Feb > Jan | first > full > new > third | high > low > falling, rising | night > day |  | | |  |
| **Black Drum** | D>B>A,C,E,F | | | Apr > Mar > Jan, Feb, May, Jun, Jul, Aug, Sep, Oct, Nov, Dec | first > full, new > third | high > falling, rising > low | night > day | 0 = 1 | | |  |
| **Oyster Toadfish** | B>A>D>C,E>F | | | Apr > May > Mar > Jun > Jul, Aug, Sep > Jan, Feb, Oct, Nov, Dec | full > third, first > new | high, rising, low > falling | day > night | **0 > 1** | | |  |
| **Silver Perch** | D>A,B,C,E>F | | | Apr > May > Mar > Jun > Jan, Feb, Jul, Aug, Sep, Oct, Nov, Dec | first, new > full, third | high > falling > rising, low | night > day | **0 > 1** | | |  |
| **Spotted Seatrout** | A,B,E >D>F,C | | | May > Jun, Jul, Aug > Sep > Apr > Oct, Nov, Dec, Jan, Feb, Mar | full > first, third > new | high > rising > falling > low | night > day | 0 = 1 | | |  |
| **Red Drum** | A,B>D>C,E,F | | | Sep > Aug > Oct > Jul, Nov, Dec, Jan, Febr, Mar, Apr, May, Jun | first > new | rising > falling, low > falling | day > night | **0 > 1** | | |  |
| **Bottlenose Dolphins** | C>A>B>E>D>F | | | Nov, Jan > Feb > Dec > Mar, Apr, Oct > May, Jun, Jul, Aug, Sep | third > full > first, new | falling > rising | night > day | **1 > 0** | | |  |

* 0 = no noise; 1 = noise present; bold and underlined values were confirmed in targeted models focused on species' calling season and circadian pattern.
